# Rapid Assignment of Chemical Shifts From Crystal Structures in Solid‐State NMR

**DOI:** 10.1002/anie.202525558

**Published:** 2026-02-24

**Authors:** Ruben Rodriguez‐Madrid, Jacob Brian Holmes, Lyndon Emsley

**Affiliations:** ^1^ Laboratory of Magnetic Resonance Institut des Sciences et Ingénierie Chimiques École Polytechnique Fédérale de Lausanne Lausanne Switzerland; ^2^ National Centre for Computational Design and Discovery of Novel Materials MARVEL Institut des Sciences et Ingénierie Chimiques École Polytechnique Fédérale de Lausanne Lausanne Switzerland

**Keywords:** Bayesian probability, chemical shift assignment, NMR crystallography, shielding predictions, solid‐state NMR

## Abstract

Chemical shift assignment in solid‐state nuclear magnetic resonance (NMR) is a challenging process that usually relies on a set of 1D and 2D experiments to determine the assignment by establishing connectivities along the covalent backbone. A Bayesian probabilistic assignment method was recently introduced based on a fragment analysis using a database of chemical shifts. Here, we propose a fast 3D structure validation method that utilizes predictions from a crystal structure as a starting point for Bayesian probabilistic chemical shift assignment. We demonstrate the approach with improved confidence in the ^1^H and ^13^C assignments for the structures of cocaine and Atuliflapon, and finally Lorlatinib which has *Z′* = 2.

## Graphical abstract

Chemical shift assignment in solids is a long and tedious process that relies on complex 1D and 2D NMR experiments. With prior knowledge of the 3D structure, this process can be significantly sped up by a Bayesian probabilistic assignment approach based on predicted chemical shifts.

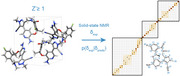

In chemical and materials sciences today, complete atomic‐level structure determination is required to develop rich models that relate structure to function. High‐resolution structures of solids are most often determined using diffraction methods [1, 2], electron microscopy [3, 4], and/or nuclear magnetic resonance (NMR) [5, 6]. NMR is particularly attractive since it does not require long‐range order, and has recently been used to solve atomic‐level structures of a range of complex materials [7, 8, 9, 10]. To determine chemically rich structures in molecular solids, NMR chemical shifts are typically combined with machine learning models and advanced computational techniques in an approach dubbed NMR‐crystallography [5, 6, 11, 12, 13]. In particular, the chemical shift is highly sensitive to hybridization, connectivity (i.e., the chemical groups bonded to a given atomic site), conformation, and the surrounding environment (i.e., hydrogen bonding, packing, solvent). It effectively encodes the local chemical environment for an atomic site, with spatial resolution in the resulting structures usually < 0.2 Å for molecular solids [14, 15].

The typical workflow consists of assigning chemical shifts to the corresponding atomic sites and comparing them with the predicted or computed chemical shifts from different candidates. The candidate whose predicted shifts best agree with the experimentally observed values is considered the correct structure [6, 16, 17].

Accurate chemical shift assignments are thus critical to the success of structure determination, but, for example, in organic solids at natural isotopic abundance this is a laborious and often challenging process. Currently employed strategies for assignment include either ^13^C‐detected experiments, involving ^13^C–^13^C correlation experiments [18, 19, 20, 21], or ^1^H‐detected workflows [22], centered around hCH type experiments [23]. In either approach the assignment process remains lengthy (days to weeks), and any tools to accelerate the process are of high value. In that context, Cordova et al. [24] introduced a general framework to aid assignments using a database of predicted chemical shifts obtained by computing shifts for 3D structures in the Cambridge Structural Database (CSD) [25]. For each atomic site in the molecule to be assigned, probability distributions of predicted chemical shifts are then constructed by matching molecular fragment descriptors centered on the atom of interest to entries in the database. The descriptors are defined as the set of covalent bonds encompassing all neighboring atoms within the local environment of the targeted site. This approach has the great advantage of only requiring knowledge of the 2D chemical structure, so it does not require the 3D structure to be known prior to assignment. However, in principle, much greater prediction accuracy could be obtained if the 3D structure is included.

Here, we propose a probabilistic approach for structure validation by using the known 3D crystal structure as a starting point, instead of the database. We demonstrate the method to assign ^13^C and ^1^H spectra of polycrystalline samples of cocaine, Atuliflapon, and Lorlatinib (*Z′* = 2).

Experimental crystal structures previously determined for cocaine [26], Atuliflapon [27], and Lorlatinib [28] were used as starting points (The coordinates for the structures are given in the Supporting Information.).

Experimental values for ^1^H and ^13^C chemical shifts have been published previously for cocaine [29], Atuliflapon [27], and Lorlatinib [28]. Quaternary and ^1^H–^13^C experimental chemical shifts were assigned from ^13^C–^13^C refocused INADEQUATE NMR and ^1^H–^13^C refocused INEPT for the case of cocaine; ^13^C–^13^C INADEQUATE and ^1^H–^13^C HETCOR for Atuliflapon; and ^1^H–^13^C HETCOR MAS and ^13^C CP MAS spectra in the case of Lornatinib. See Section S2 in the Supporting Information for all experimental chemical shifts.

Figure 1a illustrates schematically the probabilistic assignment process using the database (here dubbed DB) method to construct the probability distribution of an atomic site, for the structure of cocaine. First, a molecular graph centered at the targeted atom, in this case, C15 is constructed with a depth (*w*) determined to retain at least a statistically significant number of occurrences of that motif (*G_i_*, usually > 10) in the database. A chemical shift probability distribution is then constructed for this motif by summing the predicted chemical shifts (including their predicted uncertainties) for all the motifs. All the atoms in the database have an associated predicted chemical shift that was calculated using ShiftML2 [24, 30]. Specifically, the overall probability, *p_i_*, of observing a chemical shift value *y* ^(1)^ for a site *i* is modeled as a sum of normal Gaussian distributions (*p_k_*) centered at the predicted shifts of the different matched atomic motifs from the database *y_k_*, with its uncertainty prediction associated *σ_k_*.

(1)

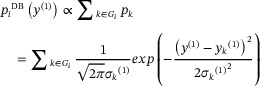




**FIGURE 1:**
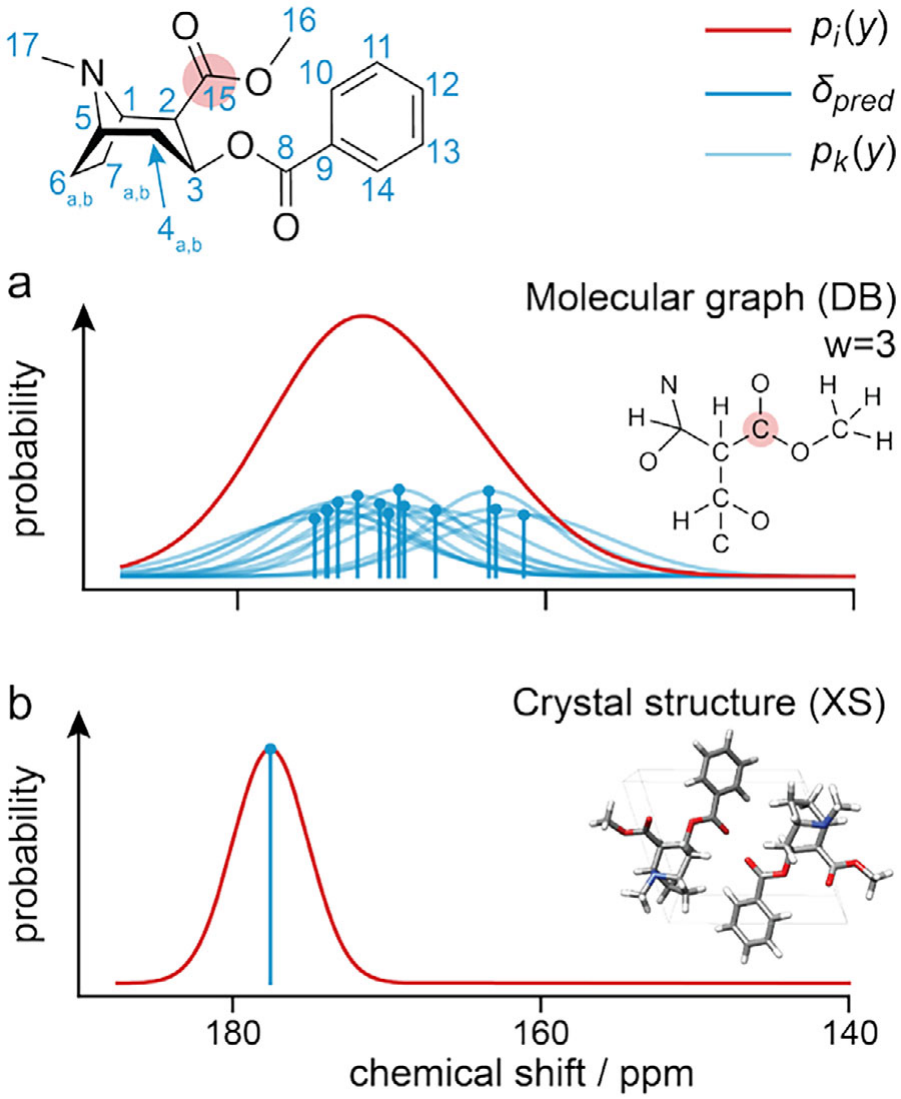
Construction of the probability distribution function for C15 of the structure of cocaine (shown inset) using the DB and XS approaches. In (a), the probability distribution *p*_i_(*y*) is constructed from a sum of Gaussian distributions (blue curves, *p*_*k*_ *(y)*) centered at the ShiftML2 predicted shifts (blue sticks) and their prediction uncertainties, for all the corresponding carbons atoms that match this graph fragment in the database (right). In (b), the probability distribution is obtained from a Gaussian centered at the shift, *y*_pred_^(1)^ *=* δ_pred_, predicted from the crystal structure, with the standard deviation given by the uncertainty in the prediction method.

Similarly, the chemical shift probability distribution of a 2D cross peak (*y* ^(1)^ *, y* ^(2)^) for a pair of correlated atoms (*i, j*) (e.g., in a C─H correlation spectrum) is given as follows:

(2)

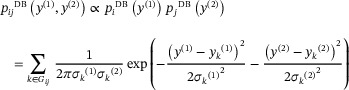

where *y* ^(1)^ and *y* ^(2)^ here correspond to the frequency domain of the first and second dimensions, respectively. Given an experimental chemical shift a*_i_*, we can then compute the conditional probability *p*(*y_j_* |*a_i_*) that it matches a particular predicted shift *y_j_* as follows:

(3) $$p\;\left(y_{j}|a_{i}\right)=\frac{p_{i}\left(y_{j}\right)}{\sum_{k}p_{i}\left(y_{k}\right)}$$

Then, given a vector of experimental chemical shifts, ***a*** (such that *a_i_ = j* if atom *i* is assigned to shift *j*), the probability that a set of predicted chemical shifts ***y*** is observed is:

(4) $$p\;\left(y|a\right)=\prod_{i}p\left(y_{a_{i}}|i\right)$$

Applying Bayes’ theorem, we can find the probability of a particular assignment ***a*** given the observed vector of shifts ***y***, considering all possible assignment permutations between predicted and experimental chemical shift vectors:

(5) $$p\;\left(a|y\right)=\frac{p\left(y|a\right)p\left(a\right)}{p\left(y\right)}=\frac{p\left(y|a\right)p\left(a\right)}{\sum_{a^{\prime }}p\left(y|a^{\prime }\right)p\left(a^{\prime }\right)}$$

where *p*(***a***) is assumed to be one, and assuming that all assignments are equally likely a priori. Finally, to get the probability that a specific atom *i* is assigned to a specific experimental peak *j*, we marginalize over all possible overall assignments:

(6) $$p\left(a_{i}=j|y\right)=\frac{\sum_{a}\delta_{a_{i}j}p\left(a|y\right)}{\sum_{a}p\left(a|y\right)}$$

where $\delta_{a_{i}j}$ is the Kronecker delta.

As mentioned above, this method has the advantage of not requiring knowledge of the 3D structure. However, if the 3D crystal structure of the molecule to be assigned is known, for example, in the case of structure validation, then the approach of Figure 1a can be adapted to capitalize on this prior knowledge, as shown in Figure 1b (here dubbed the XS approach). Here, the calculation of a distribution of possible shifts is replaced by the calculation of discrete shifts directly from the crystal structure, with an uncertainty given by the estimated uncertainty of the prediction method *σ*_pred_. In this case, Equations (1) and (2) then become as follows:

(7)

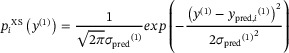

and

(8)

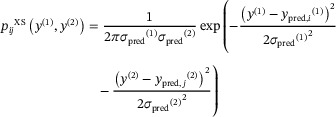

where *y*_pred_ *_,i_* ^(1)^ and *y*_pred_ *_,j_* ^(2)^ **are now the chemical shift predicted specifically for atom *i* and *j* from the known crystal structure. The rest of the process is unchanged.

The predicted shifts used as the basis for assignment can be calculated from the crystal structure using any suitable method, for example, typically for small to medium‐sized molecules, the standard approach would be GIPAW–DFT using dispersion corrected PBE functionals [31, 32]. Higher levels of theory can also be employed [33, 34, 35, 36, 37]. Here, we use the ShiftML3 machine learning model [38]. See Section S1 in the Supporting Information for more details, where we also show results using GIPAW–PBE and structures where only the H positions are geometry optimized.

For all structures, the probabilistic assignment was run for both ^1^H and ^13^C shifts using 1D (for quaternary carbons) and 2D (^1^H–^13^C) distributions, where for the 2D distributions the assignment is based on the joint probability of both the ^13^C and connected ^1^H shift. (See Supporting Information for details) ^1^H–^13^C correlation peaks were subdivided by carbon multiplicity (i.e., the number of bonded protons) during post‐processing for the case of cocaine and Atuliflapon. The case of Lorlatinib was separated into aromatic and aliphatic spectral regions.

Figure 2 shows the result of using the Bayesian probabilistic assignment of a sample of polycrystalline cocaine. A clear improvement in the confidence of the assignments is observed for almost all the atomic sites in going from the DB approach to the XS approach. For example, using the database, we see that the assignment of the H1/C1 and H5/C5 pairs remains pairwise ambiguous due to the similar chemical shifts (Δ^1^H of 0.85 ppm and Δ^13^C of 0.25 ppm) and similar molecular graphs. The predicted distribution for H5/C5 is centered at 3.20 and 59.74 ppm, and for ^1^H and ^13^C, with widths of 0.75 and 2.71, while the H1/C1 is centered at 4.05 and 59.49 ppm with widths of 0.94 and 5.12, which does not provide enough resolution to confidently distinguish the shifts, as shown in S5. When using prior knowledge of the 3D structure, these pairs of atomic sites are now confidently assigned, where the resolution is limited by the accuracy of the chemical shift prediction method (2.44 and 0.53 ppm for ^13^C and ^1^H, respectively, in the case of ShiftML3) [38]. Additionally, the XS approach has the added power to assign magnetically inequivalent atomic sites that have the same molecular graph, such as the protons of CH_2_ groups. Specifically, the database provides low confidence for assignments between the H6 and H7 protons, but H6b is confidently (and correctly) assigned to 3.38 ppm (m_1_) when using the 3D structure. This is in part due to the similarity between the molecular graphs for C6 and C7. Similarly, the aromatic CH groups 10,11,12, and 14 are highly ambiguous in the DB method, while groups 10 and 14 are confidently (and correctly) assigned in with the XS approach.

**FIGURE 2:**
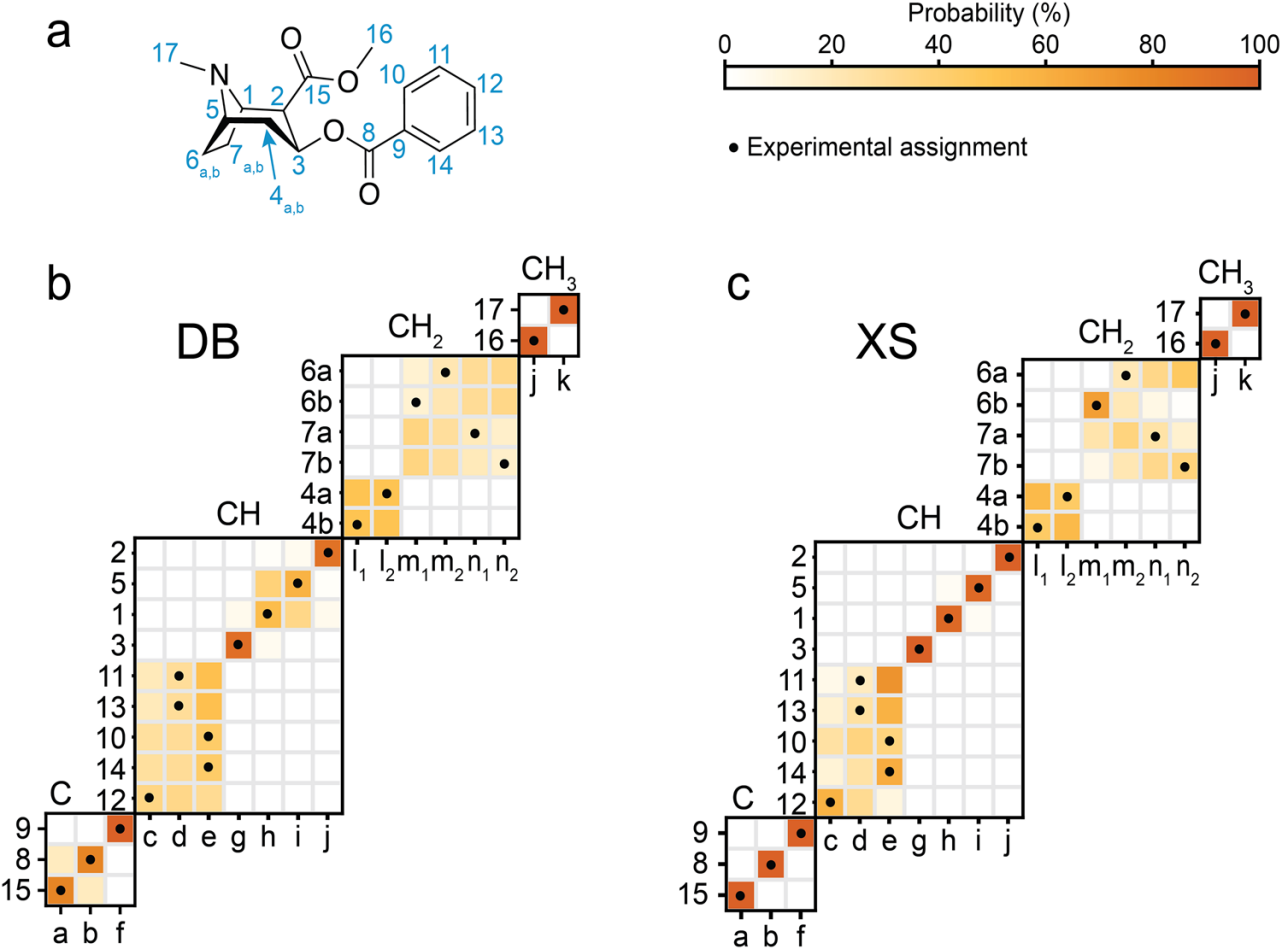
Probabilistic assignment of cocaine (a) using the (b) DB, and (c) XS approaches. Marginal individual assignment probabilities of ^1^H and ^13^C experimental chemical shifts. For each map of probable assignments, labels along the vertical axis indicate C atoms or CH atom pairs according to the numbering scheme in (a), and labels along the horizontal axis denote experimental ^13^C/^1^H shift pairs labeled alphabetically in order of decreasing ^13^C shift. The carbons have been subdivided into quaternary, tertiary, secondary, and primary, as indicated above each marginal assignment probability map. Black dots represent the experimentally determined chemical shift assignment.

Figure 3 shows the application to the assignment of Atuliflapon, which again highlights some of the advantages of incorporating prior knowledge of the 3D structure. As for cocaine, the confidence in the correct assignment across the whole spectrum is greatly increased with the XS method. Specifically looking at the six‐membered cyclohexane ring, we observe a clear improvement in the assignment with respect to the database approach. For example, in the latter one, assignments of H12/C12 and H17/C17 showed ambiguity due to the similarity of the molecular graphs scanned through the database. This is translated to chemical shift distribution differences for Δ^1^H of 0.1 ppm and Δ^13^C of 1.89 ppm, making the assignment challenging. However, incorporation of its local atomic environment (i.e., equatorial conformation of the six‐membered cyclohexane ring) on the predictions can now assign H12/C12 and H17/C17 to the pairs of experimental shifts *q* and *p* unambiguously.

**FIGURE 3:**
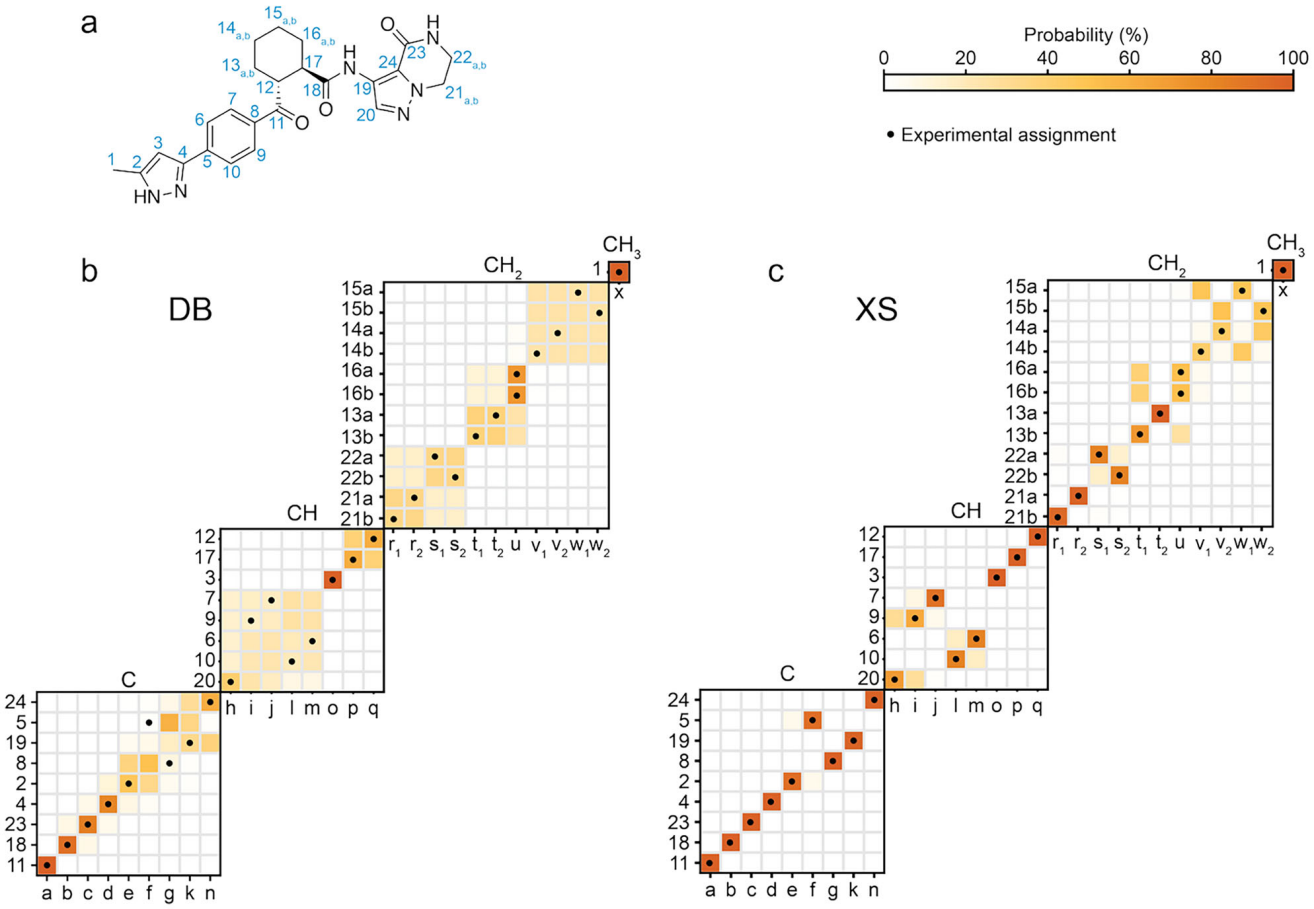
Probabilistic assignment of Atuliflapon (a) using the (b) DB, and (c) XS approaches. Marginal individual assignment probabilities of ^1^H and ^13^C experimental chemical shifts. For each map of probable assignments, labels along the vertical axis indicate C atoms or CH atom pairs according to the numbering scheme in (a), and labels along the horizontal axis denote experimental ^13^C/^1^H shift pairs labeled alphabetically in order of decreasing ^13^C shift. The carbons have been subdivided into quaternary, tertiary, secondary, and primary, as indicated above each marginal assignment probability map. Black dots represent the experimentally determined chemical shift assignment.

Further experiments, such as attaching nitrogen experiments [39], could then be used to solve remaining ambiguities, such as assignments of H20/C20 and H9/C9 to experimental chemical shifts *h* and *i* (see the Supporting Information for more information).

Overall, we see that there is a clear improvement with the XS approach. Comparing the DB and the XS approaches over all the *Z′* = 1 structures considered here, the correct assignment was found to be the most probable assignment using the XS approach for 86% of the sites, while 72% are correctly assigned using the DB approach.

Finally, we address the particularly challenging case of the free form of Lorlatinib for which the crystal structure is *Z′* = 2 [28]. The database approach is intrinsically limited to molecular crystals with *Z*′ = 1, as it cannot distinguish the pairwise uncertainty that will arise between two or more inequivalent molecules in the asymmetric unit cell (i.e., corresponding atoms in two inequivalent molecules in the unit cell will have exactly the same predicted chemical shift distribution). However, the information about inequivalent copies of the molecules is encoded in the chemical shift predictions from the 3D structure. Lorlatinib contains two molecules in the asymmetric unit cell (dubbed I and II). Peak positions with a complete experimental ^1^H and ^13^C NMR assignment for both molecules in the unit cell were proposed by Rehman et al. [28] by comparing the experimental shifts with the assigned solution‐state NMR shifts and DFT chemical shift calculations for the crystal structure.

In Figure 4, we see that the proposed probabilistic chemical shift assignment for most of the atomic sites agrees with the assignments proposed by Rehman et al. [28]. Some pairwise assignments, such as 2^I^ and 2^II^, 14^I^ and 14^II^ or 22^I^ and 22^II^ are confidently assigned, which would not have been possible with the DB approach. For others, such as 16^I^ and 16^II^, the difference between the two experimental chemical shifts is lower than the accuracy of the predictions, so that while pairwise ambiguity remains, they are in fact both confidently assigned to within error.

**FIGURE 4:**
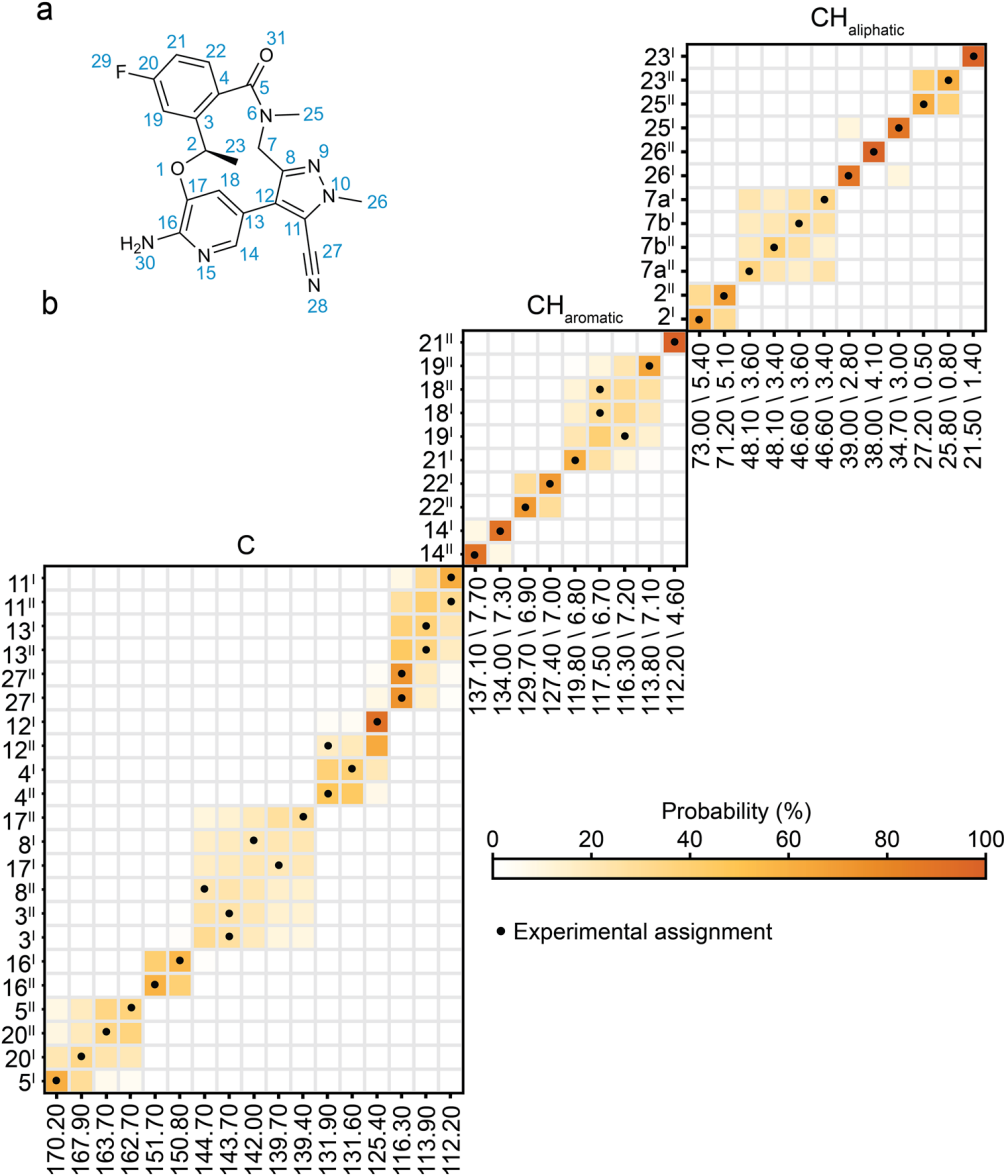
Probabilistic assignment of Lorlatinib (a) using the XS approach (b). Marginal individual assignment probabilities of ^1^H and ^13^C experimental chemical shifts. For each map of probable assignments, labels along the vertical axis indicate C atoms or CH atom pairs according to the numbering scheme in (a), together with the corresponding molecule in the asymmetric unit cell as a subscript, and labels along the horizontal axis denote experimental ^13^C/^1^H shift pairs in order of decreasing ^13^C shift. The carbons have been subdivided into quaternary, aromatic, and aliphatic, as indicated above each marginal assignment probability map. Black dots represent the experimentally determined chemical shift assignment.

On the other hand, ambiguities noted in the experimental assignments from the original paper [28], such as the four H7/C7 resonances, remain ambiguous, but now the confidence in a given assignment can be quantified in terms of probabilities. For instance, the atomic sites 7a^II^, 7b^II^, 7b^I^, and 7b^II^ are assigned to the shifts from that block (from left to right) with probabilities of 37.4%, 39.1%, 32.4%, and 34.1%.

This example also highlights how, with a large number of assignments confidently in hand, remaining ambiguities in the assignment could be resolved with targeted additional experiments, for example, for the pairwise uncertainties in atomic sites C5 and C20 by using ^19^F–^13^C CP, or the attached nitrogen test.

In summary, we have shown how incorporating prior knowledge of the 3D structure, where available, can significantly increase the accuracy of Bayesian probabilistic assignment of solid‐state NMR chemical shifts. The approach was demonstrated for ^1^H and ^13^C assignment on three challenging pharmaceutically active molecular solids, where in each case the confidence in the assignment is significantly increased. In particular, for the case of Lorlatinib, which has *Z′* = 2, an almost complete assignment of both molecules in the unit cell is proposed.

Here we have illustrated the method with ^1^H and ^13^C assignment of molecular solids, but we note that the method can be used to assign the resonances in spectra of any NMR‐active nucleus in any type of material. More generally, we remark that any improvements in the accuracy of the chemical shift calculations used as a basis for the assignment, for example, using methods to take account of finite temperature effects [40, 41, 42, 43, 44], will automatically lead to an increase in the accuracy of the method. Similarly, as demonstrated in the examples above, classifying resonances using any additional information available from complementary experiments, such as multiplicity, attached nitrogen tests, or 2D correlations, will also increase accuracy. In addition to the probabilistic framework described here, alternative approaches may also be employed to rank assignments. One such method is the DP4 analysis [45, 46, 47], which applies Bayes’ theorem using a Student's *t* distribution rather than a Gaussian model. Such a strategy can be further strengthened by explicitly integrating other experimental observables to quantitatively assess the percentage compatibility of a given assignment, as proposed by Evans to resolve two‐ three‐, or four‐way ambiguities in assignment [48].

We finally note that the probabilistic methods discussed here are not intended to completely replace experiments to determine assignments. Rather, they serve as a basis to accelerate assignment, for example, by directing the spectroscopist to the resonances that are most ambiguous, and thereby guiding the choice of complementary experiments required to complete the assignment, as necessary.

## Conflicts of Interest

The authors declare no conflicts of interest.

## Supporting information




**Supporting File 1**: Details about the geometry optimization, predicted shieldings, and experimental chemical shifts, and other probabilistic assignments (i.e., using DFT shieldings, attached nitrogen carbons for Atuliflapon) are described in the Supporting Information. Source code, raw input, and output files can be found in the following link: https://zenodo.org/uploads/17487125 (will be activated on publication).
**Supporting File 2**: anie71582‐sup‐0001‐SuppMat.pdf.

## Data Availability

The data that support the findings of this study are openly available in Zenodo at https://doi.org/[on_acceptance], reference number 123456.
