# Supplementary material for: Rapid Assignment of Chemical Shifts From Crystal Structures in Solid‐State NMR
Source: Angew Chem Int Ed Engl. 2026 Feb 24;65(14):e25558. doi: 10.1002/anie.202525558 (PMC13023724; doi:10.1002/anie.202525558)
Supplement: Supplementary file 1 — Supporting File 1: Details about the geometry optimization, predicted shieldings, and experimental chemical shifts, and other probabilistic assignments (i.e., using DFT shieldings, attached nitrogen carbons for Atuliflapon) are described in the Supporting Information. Source code, raw input, and output files can be found in the following link: https://zenodo.org/uploads/17487125 (will be activated on publication). Supporting File 2: anie71582‐sup‐0001‐SuppMat.pdf. [file ANIE-65-e25558-s001.pdf]

Supporting Information for

Rapid Assignment of Chemical Shifts from Crystal Structures in Solid-State NMR

Ruben Rodriguez-Madrid,[a] Jacob Brian Holmes[a,b] and Lyndon Emsley*[a,b]

[a] R. Rodriguez-Madrid, J. B. Holmes, L. Emsley
Laboratory of Magnetic Resonance
Institut des Sciences et Ingénierie Chimiques, École Polytechnique Fédérale de Lausanne
1015 Lausanne, Switzerland
E-mail: lyndon.emsley@epfl.ch

[b] J. B. Holmes, L. Emsley
National Centre for Computational Design and Discovery of Novel Materials MARVEL
Institut des Sciences et Ingénierie Chimiques, École Polytechnique Fédérale de Lausanne
1015 Lausanne, Switzerland

Table of Contents

S1. NMR chemical shift calculations and referencing 2
S2. Tables of experimental chemical shifts together with shielding predictions using DFT and sML3 3
2.1. Cocaine 3
2.2. Atuliflapon 4
2.3. Lorlatinib 5
S3. ^{1}H and ^{13}C linear regression between experimental and predicted shielding for Atuliflapon 6
S4. Other Bayesian Probabilistic assignments 7
4.1. Cocaine using DFT shifts 7
4.2. Cocaine using ShiftML shifts on an only-proton relaxed structure 8
4.3. Atuliflapon with DFT shifts 9
4.3.1. Resolving ambiguity using knowledge of an attached nitrogen 9
4.4. Lorlatinib with DFT shifts 11
4.4.1. Resolving ambiguity using knowledge of an attached nitrogen 12
S5. Chemical shift distributions for the probabilistic assignment 13
5.1. Construction of the chemical shift distributions for the DB approach 13
5.1.1. Cocaine 13
5.1.2. Atuliflapon 13
5.2. Chemical shift distributions and experimental chemical shifts 14
5.2.1. Cocaine 14
5.2.2. Atuliflapon 17
5.2.3. Lorlatinib 20
References 22

S1. NMR chemical shift calculations and referencing

DFT chemical shift calculations were done using the Quantum ESPRESSO (QE) v7.2[1, 2] package for geometry optimization and GIPAW NMR calculations. A Monkhorst−Pack K-point grid[3] with a maximum spacing of 0.06 $Å^{-1}$ was used for both geometry optimization and shielding calculations.

Geometry optimization of all atoms for cocaine and Atuliflapon was done using the PBE density functional[4, 5] and the Grimme D3 dispersion correction,[6, 7] keeping the cell parameters fixed. Wavefunction and charge density energy cut-offs of 100 and 400 Ry, respectively. The same process was used to only relax protons for the structure of cocaine. For the case of Lorlatinib, PBE density functionals with Grimme D2 corrections were used with wave-function and charge dentity energy cut-offs of 60 and 240 Ry, respectively. For all structures ultrasoft pseudopotentials H.pbe-tm-new-gipaw-dc.UPF, C.pbe-tm-new-gipaw-dc.UPF, N.pbe-n-kjpaw_psl.1.0.0.UPF, O.pbe-n-kjpaw_psl.1.0.0.UPF and F.pbe-n-kjpaw_psl.1.0.0.UPF were used from http://www.quantum-espresso.org.

Note that for Lorlatinib we used the D2 dispersion correction because the error in the DFT predicted shifts with respect to the experimental chemical shifts of most of the quaternary carbons, in particular sites 5 and 20, is significantly worse with D3 as compared to the predictions using the D2 geometries. (The RMSD between predictions and experiment is 2.1 ppm for all carbons with D2, compared to 2.6 ppm for all carbons with D3: this change is well outside the error margins). We have not investigated the origin of this specific error.

DFT shielding calculations were run using GIPAW and same DFT parameters as for structure relaxation, increasing the wavefunction and charge density energy cut-offs to 100 and 400 Ry, respectively, for the case of Lorlatinib. Ultrasoft pseudopotentials with GIPAW reconstruction from the USSP pseudopotential database v1.0.0, H.pbe-kjpaw_psl.1.0.0.UPF, C.pbe-n-kjpaw_psl.1.0.0.UPF, N.pbe-n-kjpaw_psl.1.0.0.UPF, O.pbe-n-kjpaw_psl.1.0.0.UPF and F.pbe-n-kjpaw_psl.1.0.0.UPF were used. For cocaine and Lorlatinib, the same linear transformation (slope/intercept) as used in ShiftML3[8] was used to transform shielding to chemical shifts to evaluate the experimental benchmark. For DFT calculation, (-0.8902/27.58) was used for ^{1}H and (-0.9736/166.06) for ^{13}C.

For ShiftML3 (sML3) chemical shift predictions the DFT geometry optimized structures were taken as starting point for shielding calculations. Snippets of how to run those calculations using the ASE interface on a Python code are well described in https://github.com/lab-cosmo/ShiftML For ShiftML3, linear transformations from shieldings to chemical shifts (slope/intercept) were done using (-0.9024/28.05) for ^{1}H and (-0.9732/166.23) for ^{13}C for cocaine and Lorlatinib.

Experimental chemical shift assignments for Atuliflapon were referenced with respect to a different reference compound than the one used in the experimental benchmark. Hence, the linear regression described in Section S3 is used. In Table 1, the RMSEs given for sML3 and DFT are the standard deviation of the normal Gaussian distributions centered at the prediction peak used to run the probabilistic assignment.

Ambiguous assignments of experimental chemical shifts were solved by selecting the assignment yielding the lowest shift RMSE.

Input and output files can be found in https://zenodo.org/uploads/17487125

Table 1. Standard deviations (defined as the RMSE of the shielding prediction method used) considered for the construction of the normalized Gaussian distributions centered at the predicted chemical shift for ^{1}H and ^{13}C obtained from the experimental benchmark published in [8].

Nucleus	XS Method	σ/RMSE [ppm]
^{1}H	DFT	0.49
	sML3	0.53
^{13}C	DFT	2.34
	sML3	2.44

S2. Tables of experimental chemical shifts together with shielding predictions using DFT and sML3

2.1. Cocaine

	Experimental chemical shifts [ppm][9]		Experimental chemical shift	DFT shieldings		sML3 shieldings		sML3 shieldings * Only H-relaxed structure	
Label	^{13}C	^{1}H	Label[10]	^{13}C	^{1}H	^{13}C	^{1}H	^{13}C	^{1}H
1	65.95	3.76	h	98.92	27.09	101.65	26.93	102.19	26.99
2	50.16	3.78	j	119.29	26.88	120.7	27.28	121.46	27.26
3	66.70	5.63	g	98.04	25.13	98.61	25.33	99.87	25.41
4	36.66	3.06, 3.32	l (l1, l2)	135.50	27.80, 28.26	135.39	28.17, 28.37	135.98	28.11, 28.36
5	62.63	3.49	i	104.52	27.24	106.41	27.34	107.02	27.36
6	25.62	3.38, 2.91	m (m1, m2)	147.66	27.71, 28.70	151.01	28.23, 29.00	151.20	28.31, 29.01
7	25.62	2.56, 2.12	n (n1, n2)	145.99	28.44, 28.83	150.05	28.64, 28.96	150.25	28.66, 28.96
8	165.94	–	b	-5.54	-	-1.55	-	0.32	-
9	129.37	–	f	37.81	-	38.89	-	38.86	-
10	131.5	8.01	e	35.31	22.43	36.72	22.54	36.96	22.56
11	133.5	8.01	d	39.38	22.81	40.08	23.05	40.03	23.04
12	134.53	8.01	c	31.69	22.28	33.44	22.87	33.72	22.89
13	133.5	8.01	d	36.46	22.13	38.59	22.74	38.71	22.77
14	131.5	8.01	e	38.21	22.45	38.79	22.82	38.77	22.84
15	172.18	–	a	-13.81	-	-10.6	-	-8.89	-
16	50.16	3.78	j	118.1	26.9	116.92	26.92	117.63	26.9
17	41.52	1.04	k	129.42	29.72	131.56	29.48	131.68	29.5

2.2. Atuliflapon

	Experimental chemical shifts [ppm][11]		Experimental chemical shifts	DFT shieldings		sML3 shieldings	
Label	^{13}C	^{1}H	Label[10]	^{13}C	^{1}H	^{13}C	^{1}H
1	11.1	1.2	x	159.99	29.25	161.23	29.36
2	141.5	-	e	22.59	-	23.56	-
3	102.3	5.8	o	61.51	23.73	64.75	23.78
4	149.8	-	d	14.48	-	14.34	-
5	139.5	-	f	26.32	-	31.59	-
6[a]	123.9	6.9	m	42.54	22.88	44.53	23.87
7[b]	130.1	6.7	j	36.3	23.49	39.16	24.38
8	133.3	-	g	35.46	-	35.44	-
9[b]	130.8	7.0	i	34.04	22.27	36.10	22.79
10[a]	125.3	7.3	j	44.08	23.09	43.21	23.07
11	201.1	-	a	-43.13	-	-40.87	-
12	46.3	3.9	q	122.8	26.13	121.67	26.93
13	31.2	1.7, 0.0	t (t_1, t_2)	138.61	28.55, 30.39	137.92	28.72, 30.42
14	26.6	0.8, -0.5	v (v_1, v_2)	145.4	29.47, 30.02	146.11	29.58, 29.99
15	26.0	0.8, -0.5	w (w_1, w_2)	145.25	29.37, 30.81	145.77	29.22, 30.65
16	29.2	1.6	u	142.13	28.45, 29.07	140.31	28.93, 28.95
17	49.8	1.6	p	120.34	28.84	121.37	28.70
18	174.0	-	b	-11.35	-	-6.34	-
19	125.8	-	k	38.43	-	40.55	-
20[b]	130.8	7.6	h	32.23	22.37	34.03	22.45
21	43.5	2.7, 1.7	r (r_1, r_2)	126.48	27.58, 28.85	126.46	27.87, 29.60
22	40.1	2.7, 1.9	s (s_1, s_2)	132.32	27.32, 28.11	132.99	27.58, 28.13
23	161.8	-	c	4.12	-	7.95	-
24	119.7	-	n	45.03	-	45.68	-

† The values for inequivalent protons attached to the same carbon (CH_2) are indicated by a comma, and groups of ambiguous assignments are denoted by superscripts on the atomic site label.

2.3. Lorlatinib

	Experimental chemical shifts [ppm][12]		DFT shieldings		sML3 shieldings	
**Label**	**^{13}C**	**^{1}H**	**^{13}C**	**^{1}H**	**^{13}C**	**^{1}H**
2	71.2^{II}, 73.0^{I}	5.4^{I}, 5.1^{II}	95.62^{II}, 93.19^{I}	25.28^{I}, 25.77^{II}	94.64^{II}, 93.30^{I}	25.49^{I}, 25.81^{II}
3	142.0		23.95 ^{I}, 24.0 ^{II}	-	22.69^{I}, 23.23^{II}	-
4	125.4^{I}/131.6^{I}, 131.9^{II}	-	37.03^{I}, 36.06^{II}	-	39.94^{I}, 38.96^{II}	-
5	162.7^{II}/163.7^{II} 167.9^{I}/170.2^{I}	-	-0.33^{II}, -4.02^{I}	-	-0.94^{II}, -3.42^{I}	-
7a	46.6^{I},48.1^{II}	3.6	123.72^{I}, 121.76^{II}	26.86	124.15^{I}, 122.05^{II}	26.76
7b	46.6^{I},48.1^{II}	3.4	123.72^{I}, 121.76^{II}	26.86	124.15^{I}, 122.05^{II}	27.07
8	143.7^{I}, 144.7^{II}	-	25.62^{I}, 22.1^{II}	-	24.11^{I}, 23.42^{II}	-
11	112.2	-	56.20^{I}, 55.90^{II}	-	55.3^{I}, 52.90^{II}	-
12	125.4^{I}/131.6^{I}, 131.9^{II}	-	37.35^{I}, 35.22^{II}	-	42.81^{I}, 41.13^{II}	-
13	116.3	-	54.21^{I}, 53.05^{II}	-	51.97^{I}, 51.44^{II}	-
14	134.0^{I}, 137.1^{II}	7.3^{I}, 7.7^{II}	33.74^{I}, 29.56^{II}	23.27^{I}, 22.83^{II}	30.70^{I}, 28.52^{II}	23.18^{I}, 22.45^{II}
16	150.8^{I}, 151.7^{II}	-	18.68^{I}, 16.38^{II}	-	19.92^{I}, 17.76^{II}	-
17	139.4^{II}, 139.7^{I}	-	26.76^{II}, 25.84^{I}	-	24.83^{II}, 24.01^{I}	-
18	117.5	6.7	49.80^{I}, 51.78^{II}	23.98^{I}, 24.25^{II}	52.28^{I}, 53.81^{II}	23.81^{I}, 23.92^{II}
19	113.8^{II}, 116.3^{I}	7.2^{I}, 7.1^{II}	54.57^{II}, 50.34^{I}	23.54^{I}, 23.58^{II}	54.82^{II}, 51.89^{I}	24.01^{I}, 23.74^{II}
20	167.9 ^{I}/170.2 ^{I}, 162.7 ^{II}/163.7^{II}	-	-2.99 ^{I}, -2.47^{II}	-	-1.84^{I}, -0.99^{II}	-
21	112.2^{II} 119.8^{I}	6.8^{I},4.6^{II}	56.93^{II}, 49.39^{I}	23.57^{I}, 25.61^{II}	54.47^{II}, 49.68^{I}	23.96^{I}, 25.87^{II}
22	127.4^{I}, 129.7^{II}	7.0^{I}, 6.9^{II}	40.29^{I}, 37.69^{II}	23.42^{I}, 23.77^{II}	40.69^{I}, 38.79^{II}	23.56^{I}, 23.94^{II}
23	21.5^{I}, 25.8^{II}	1.4^{I}, 0.8^{II}	152.94^{I}, 147.17^{II}	29.55^{I}, 30.11^{II}	152.29^{I}, 145.96^{II}	29.85^{I}, 29.91^{II}
25	27.2^{II}, 34.7^{I}	3.0^{I}, 0.5^{II}	145.77^{II}, 135.87^{I}	27.79^{I}, 29.99^{II}	144.34^{II}, 135.01^{I}	27.77^{I}, 29.95^{II}
26[a]	38.0^{II}, 39.0^{I}	2.8^{I}, 4.1^{II}	133.53^{II}, 131.35^{I}	27.79^{I}, 26.29^{II}	134.44^{II}, 132.42^{I}	28.04^{I}, 26.7^{II}
27	113.9	-	49.54^{I}, 48.86^{II}	-	48.80^{I}, 48.64^{II}	-

† The values for inequivalent protons attached to the same carbon (CH_2) are indicated by a comma, and a slash denotes ambiguous assignments. Next to each experimental chemical shift/shielding, there is the denoted in a superscript the molecule it corresponds to in the asymmetric unit cell (i.e., molecule I or II)

S3. [1]H and ^{13}C linear regression between experimental and predicted shielding for Atuliflapon

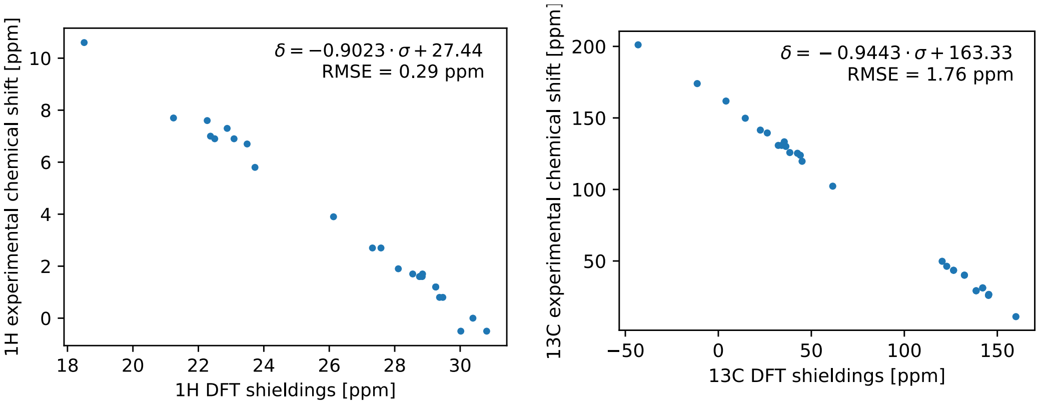

Figure S 1. Linear regression for the structure of Atuliflapon between experimental and computed DFT shieldings, for ^{1}H and ^{13}C, respectively.

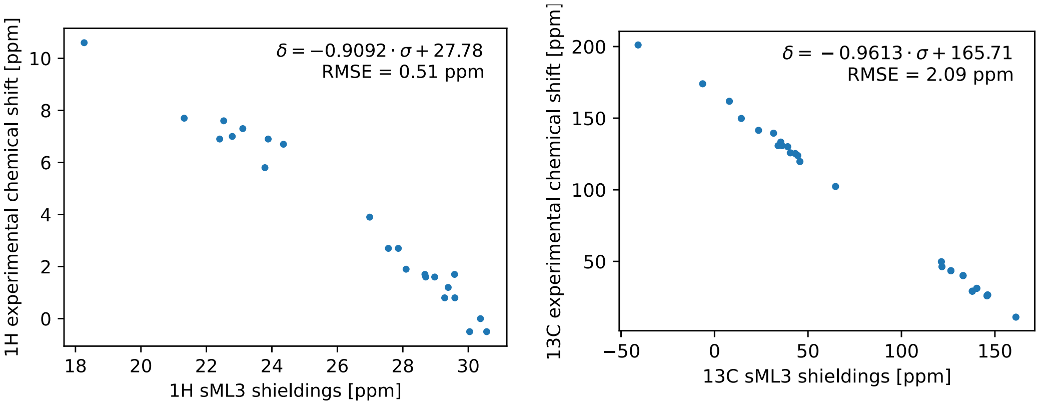

Figure S 2. Linear regression for the structure of Atuliflapon between experimental and computed sML3 shieldings, for ^{1}H and ^{13}C, respectively.

S4. Other Bayesian Probabilistic assignments

4.1. Cocaine using DFT shifts

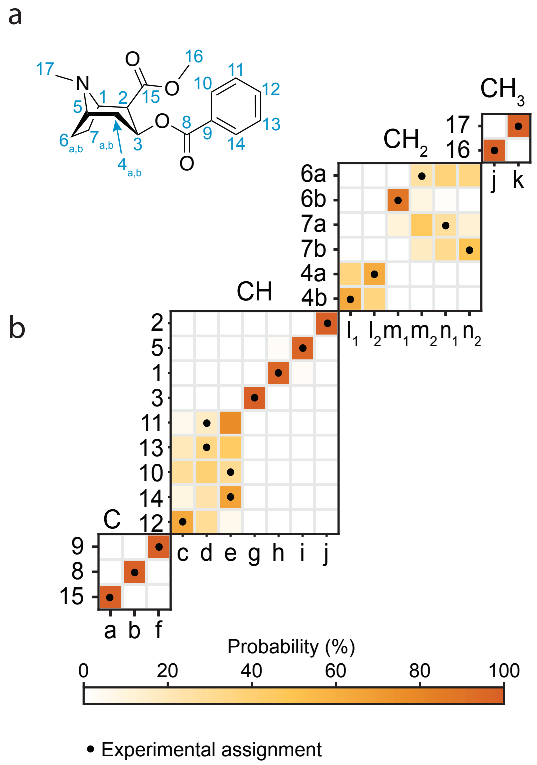

Figure S 2. Probabilistic assignment of cocaine (a) using the (b) XS approach. Marginal individual assignment probabilities of ^{1}H and ^{13}C experimental chemical shifts. For each map of probable assignments, labels along the vertical axis indicate C atoms or CH atom pairs according to the numbering scheme in (a), and labels along the horizontal axis denote experimental ^{13}C / ^{1}H shift pairs shifts labeled alphabetically in order of decreasing ^{13}C shift. The carbons have been sub-divided into quaternary, tertiary, secondary, and primary, as indicated above each marginal assignment probability map. Black dots represent the experimentally determined chemical shift assignment.

4.2. Cocaine using ShiftML shifts on an only-proton relaxed structure

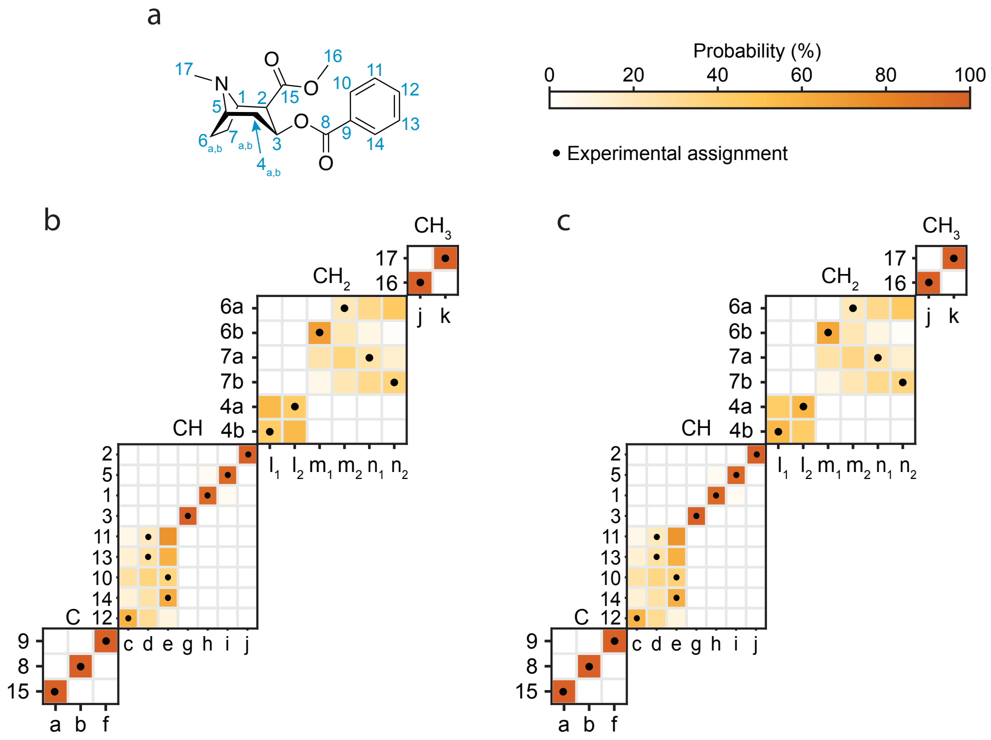

Figure S 3. Probabilistic assignment of cocaine (a) using the XS approach on the (b) all relaxed atoms and (c) only-proton relaxed XRD structure. Marginal individual assignment probabilities of ^{1}H and ^{13}C experimental chemical shifts. For each map of probable assignments, labels along the vertical axis indicate C atoms or CH atom pairs according to the numbering scheme in (a), and labels along the horizontal axis denote experimental ^{13}C / ^{1}H shift pairs shifts labeled alphabetically in order of decreasing ^{13}C shift. The carbons have been sub-divided into quaternary, tertiary, secondary, and primary, as indicated above each marginal assignment probability map. Black dots represent the experimentally determined chemical shift assignment.

4.3. Atuliflapon with DFT shifts

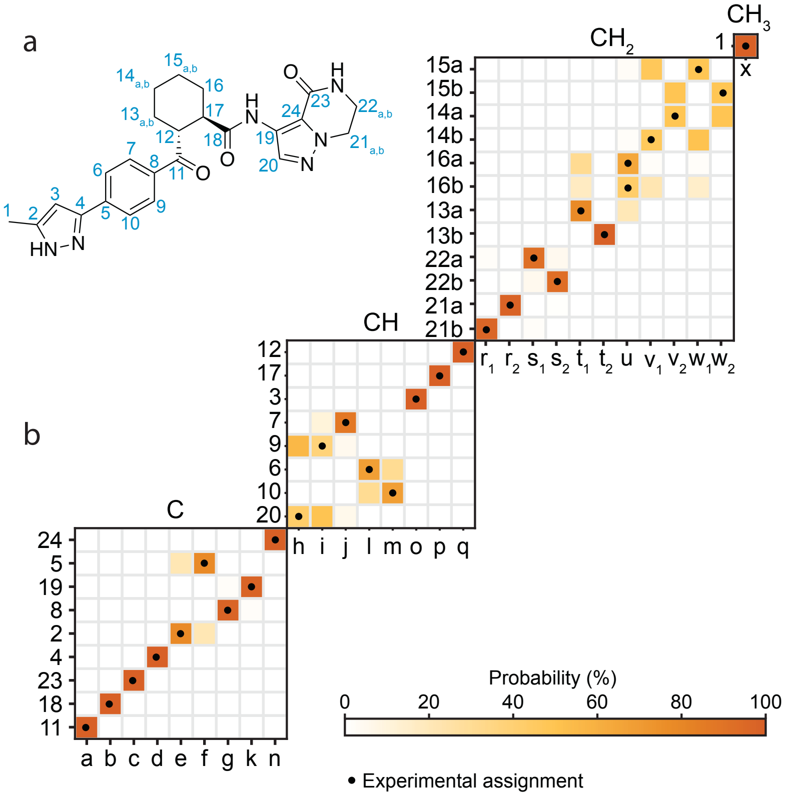

Figure S 4. Probabilistic assignment of Atuliflapon (a) using the (b) XS approach. Marginal individual assignment probabilities of ^{1}H and ^{13}C experimental chemical shifts. For each map of probable assignments, labels along the vertical axis indicate C atoms or CH atom pairs according to the numbering scheme in (a), and labels along the horizontal axis denote experimental ^{13}C / ^{1}H shift pairs labeled alphabetically in order of decreasing ^{13}C shift. The carbons have been sub-divided into quaternary, tertiary, secondary, and primary, as indicated above each marginal assignment probability map. Black dots represent the experimentally determined chemical shift assignment.

4.3.1. Resolving ambiguity using knowledge of an attached nitrogen

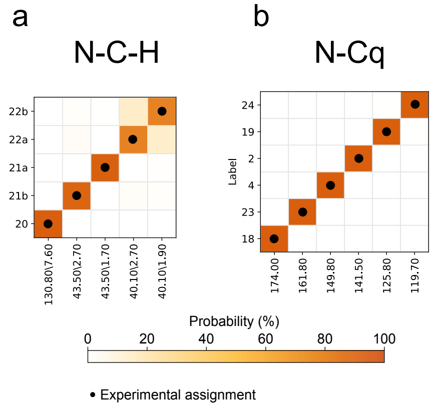

Figure S 5. Probabilistic assignment of (a) protonated and (b) quaternary carbons attached to a nitrogen in Atuliflapon using the XS approach with the sML3 predictions. Marginal individual assignment probabilities of ^{1}H and ^{13}C experimental chemical shifts. For each map of probable assignments, labels along the vertical axis indicate C atoms or CH atom pairs according to the numbering scheme in the main text, and labels along the horizontal axis denote experimental ^{13}C / ^{1}H shift pairs in order of decreasing ^{13}C shift. Black dots represent the experimentally determined chemical shift assignment.

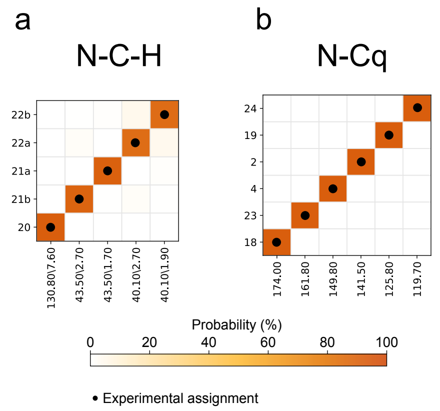

Figure S 6. Probabilistic assignment of (a) protonated and (b) quaternary carbons attached to a nitrogen in Atuliflapon using the XS approach with the DFT predictions. Marginal individual assignment probabilities of ^{1}H and ^{13}C experimental chemical shifts. For each map of probable assignments, labels along the vertical axis indicate C atoms or CH atom pairs according to the numbering scheme in the main text, and labels along the horizontal axis denote experimental ^{13}C / ^{1}H shift pairs in order of decreasing ^{13}C shift. Black dots represent the experimentally determined chemical shift assignment.

4.4. Lorlatinib with DFT shifts

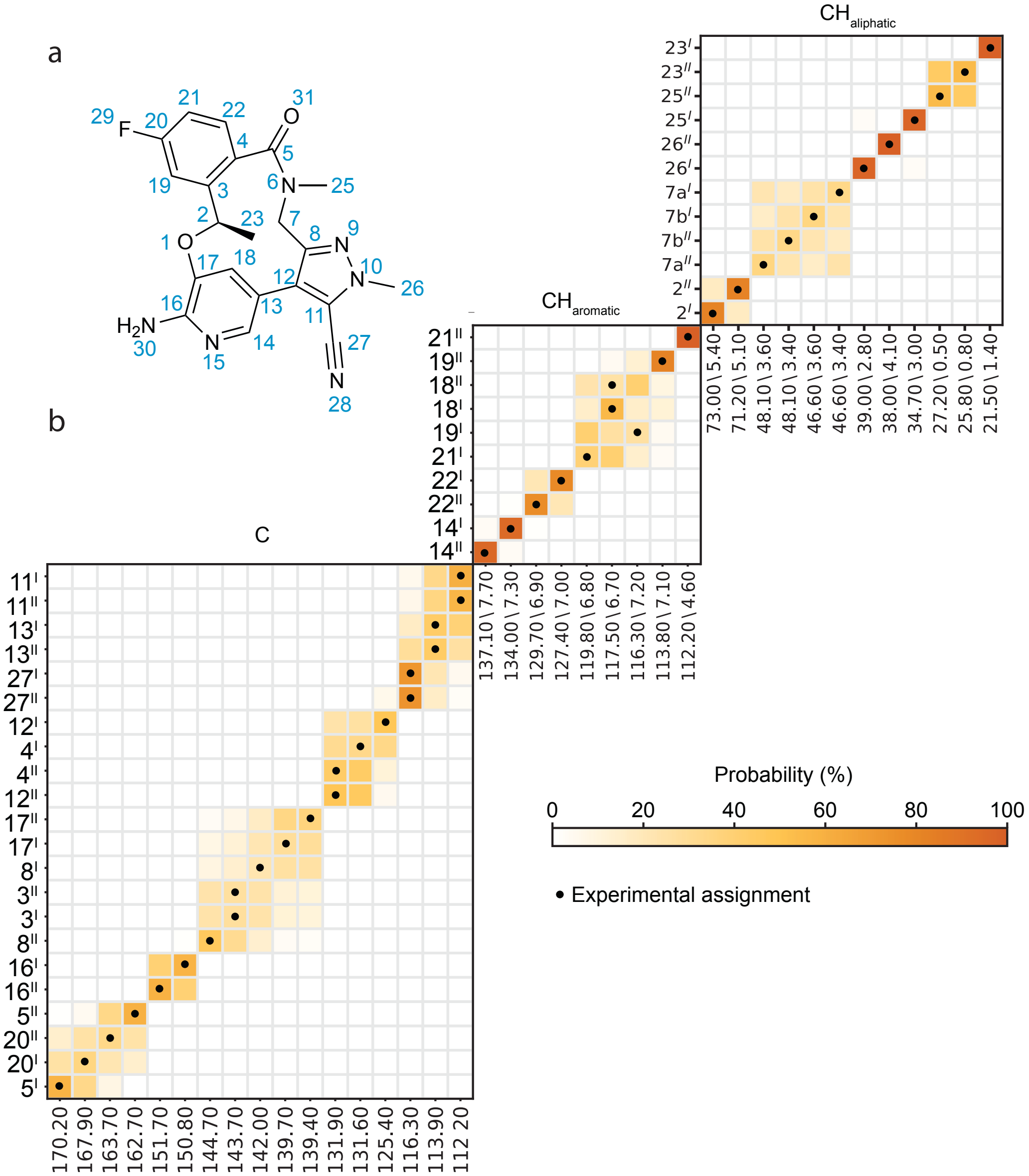

Figure S 7. Probabilistic assignment of Lorlatinib (a) using the (b) XS approach. Marginal individual assignment probabilities of ^{1}H and ^{13}C experimental chemical shifts. For each probability map, labels along the vertical axis indicate C atoms or CH atom pairs according to the numbering scheme in (a), together with the corresponding molecule in the asymmetric unit cell as a subscript, and labels along the horizontal axis denote experimental ^{13}C / ^{1}H shift pairs in order of decreasing ^{13}C shift. The carbons have been sub-divided into quaternary, aromatic, and aliphatic, as indicated above each marginal assignment probability map. Black dots represent the experimentally determined chemical shift assignment.

4.4.1. Resolving ambiguity using knowledge of an attached nitrogen

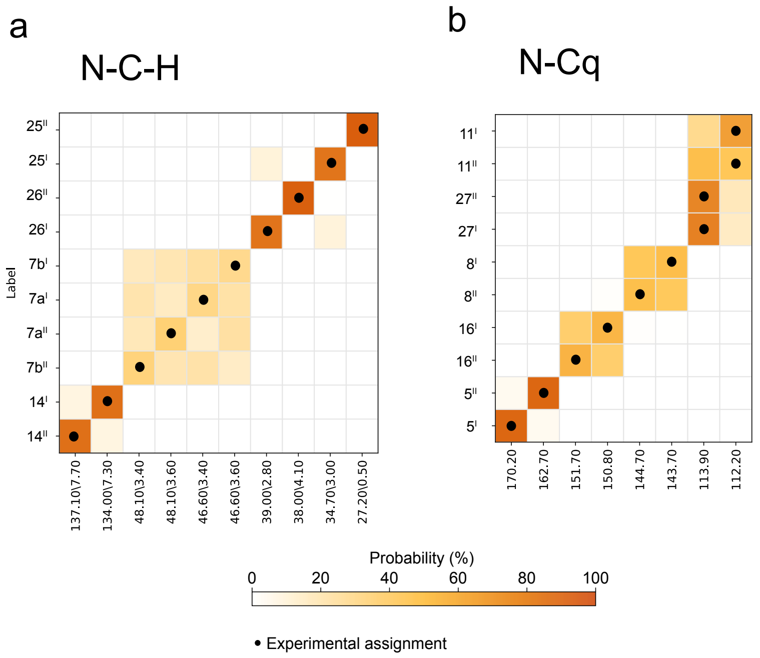

Figure S 8. Probabilistic assignment of (a) protonated and (b) quaternary carbons attached to a nitrogen in Lorlatinib using the XS approach with the sML3 predictions. Marginal individual assignment probabilities of ^{13}C experimental chemical shifts 2D correlated experiments. For each map of probable assignments, labels along the vertical axis indicate nuclei according to the numbering scheme in the structure, and labels along the horizontal axis denote experimental shifts labeled alphabetically in order of decreasing ^{13}C shift.

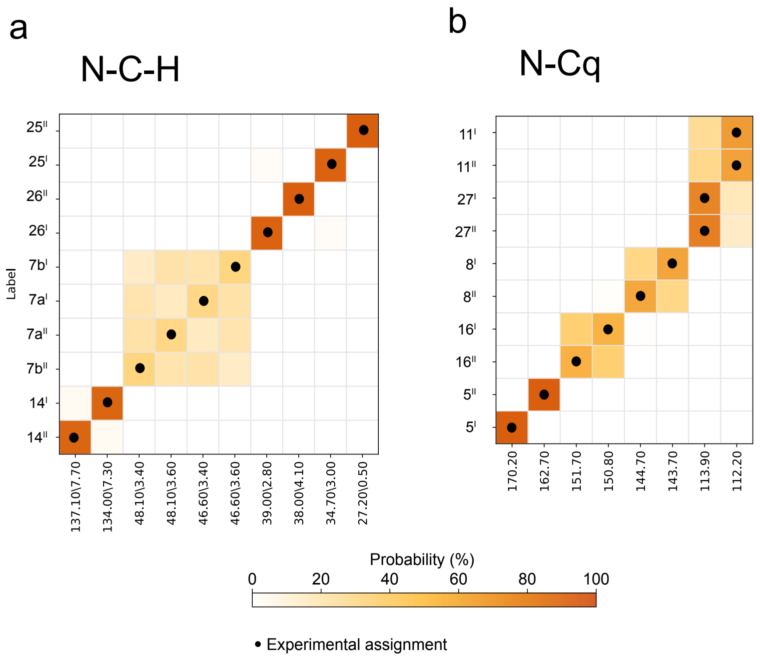

Figure S 9. Probabilistic assignment of (a) protonated and (b) quaternary carbons attached to a nitrogen in Lorlatinib using the XS approach with the DFT predictions. Marginal individual assignment probabilities of ^{13}C experimental chemical shifts 2D correlated experiments. For each map of probable assignments, labels along the vertical axis indicate nuclei according to the numbering scheme in the structure, and labels along the horizontal axis denote experimental shifts labeled alphabetically in order of decreasing ^{13}C shift.

S5. Chemical shift distributions for the probabilistic assignment

5.1. Construction of the chemical shift distributions for the DB approach

The following 2D (x = ^{13}C, y = ^{1}H) normalized distributions (in blue) are examples constructed from the instances (N) that match the molecular fragment w-bonds depth of the targeted atomic site by screening through the ShiftML2 database of structures. For each dimension, in subscripts, the center of the projected Gaussian (μ) and its standard deviation (σ) are specified.

5.1.1. Cocaine

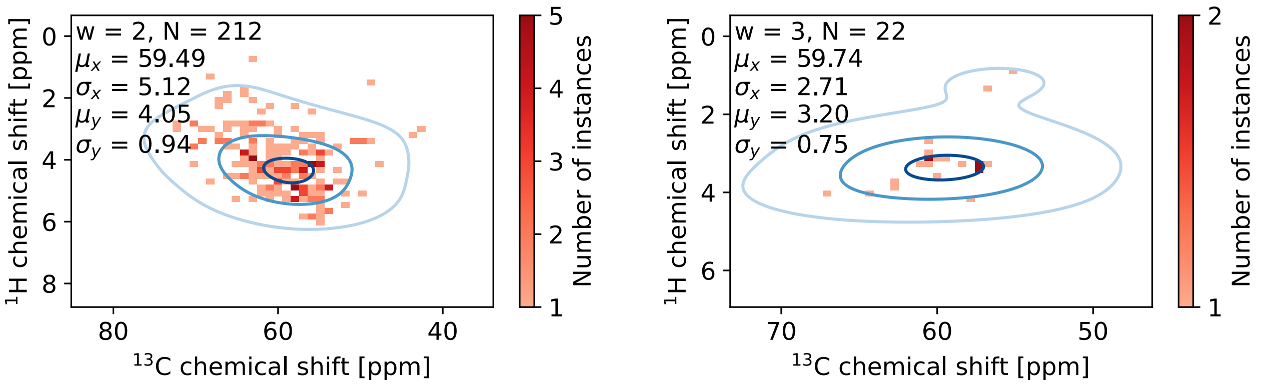

Figure S 10. Chemical shift distributions of H1/C1 and H5/C5, respectively, for cocaine screened from the ShiftML database.

5.1.2. Atuliflapon

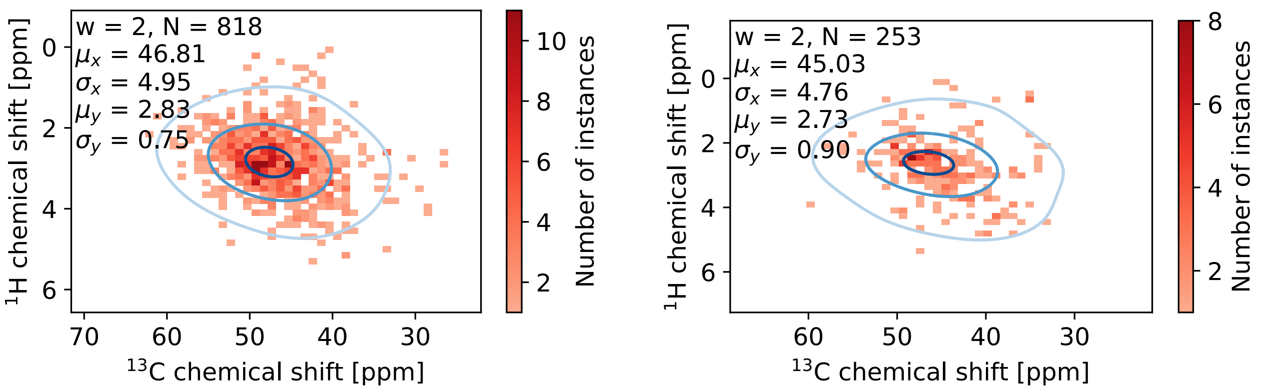

Figure S 11. Chemical shift distributions of H12/C12 and H17/C17, respectively, for Atuliflapon screened from the ShiftML database.

From the normalized distributions constructure form the hits of the ShiftML database, we can run the probabilistic assignment as described in the main text.

5.2. Chemical shift distributions and experimental chemical shifts

Distributions from the DB and XS approaches together with the experimental chemical shifts (black dots) for the 2D ^{1}H-^{13}C correlation peaks and the quaternary ^{13}C.

5.2.1. Cocaine

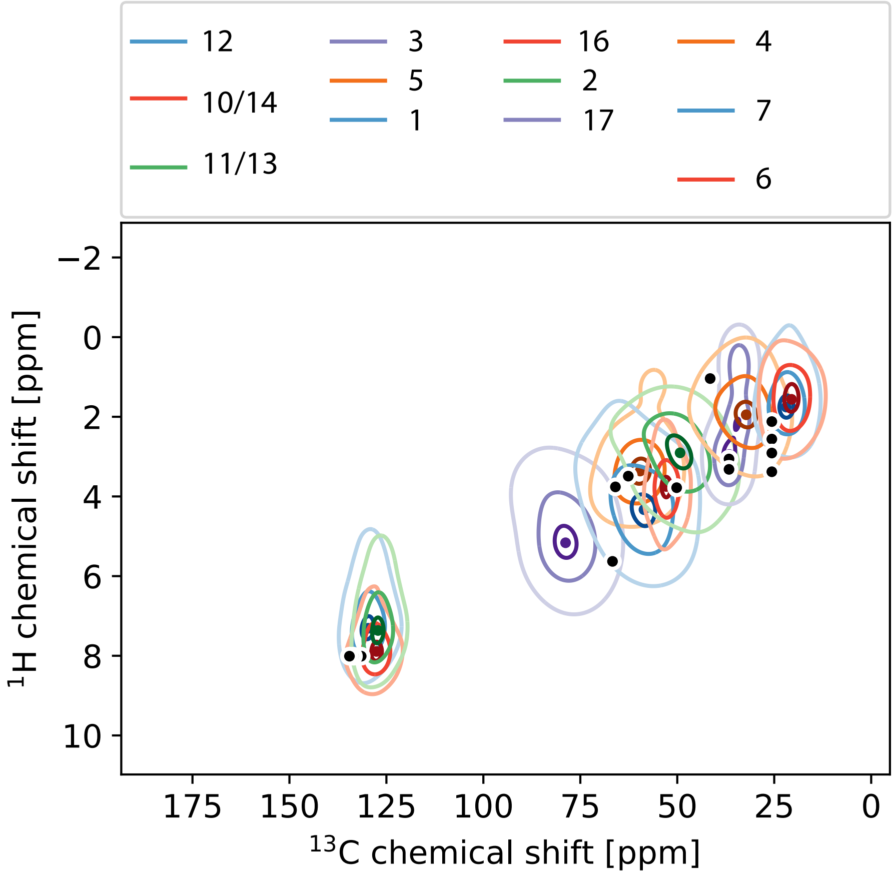

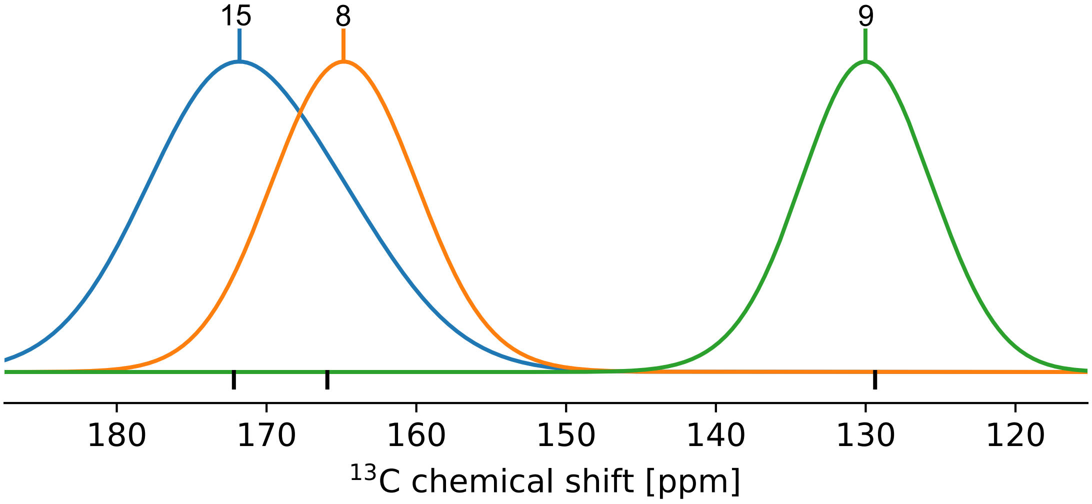

Figure S 12. Chemical shift distributions for cocaine using the DB approach. 2D ^{1}H-^{13}C correlated peaks and quaternary ^{13}C spectra, respectively, are colored with the respective labels in the legend, and experimental shifts are indicated by black dots in the correlated ^{1}H-^{13}C chemical shift distributions (upper), as well as by black vertical lines under the 1D ^{13}C chemical shift distributions (lower).

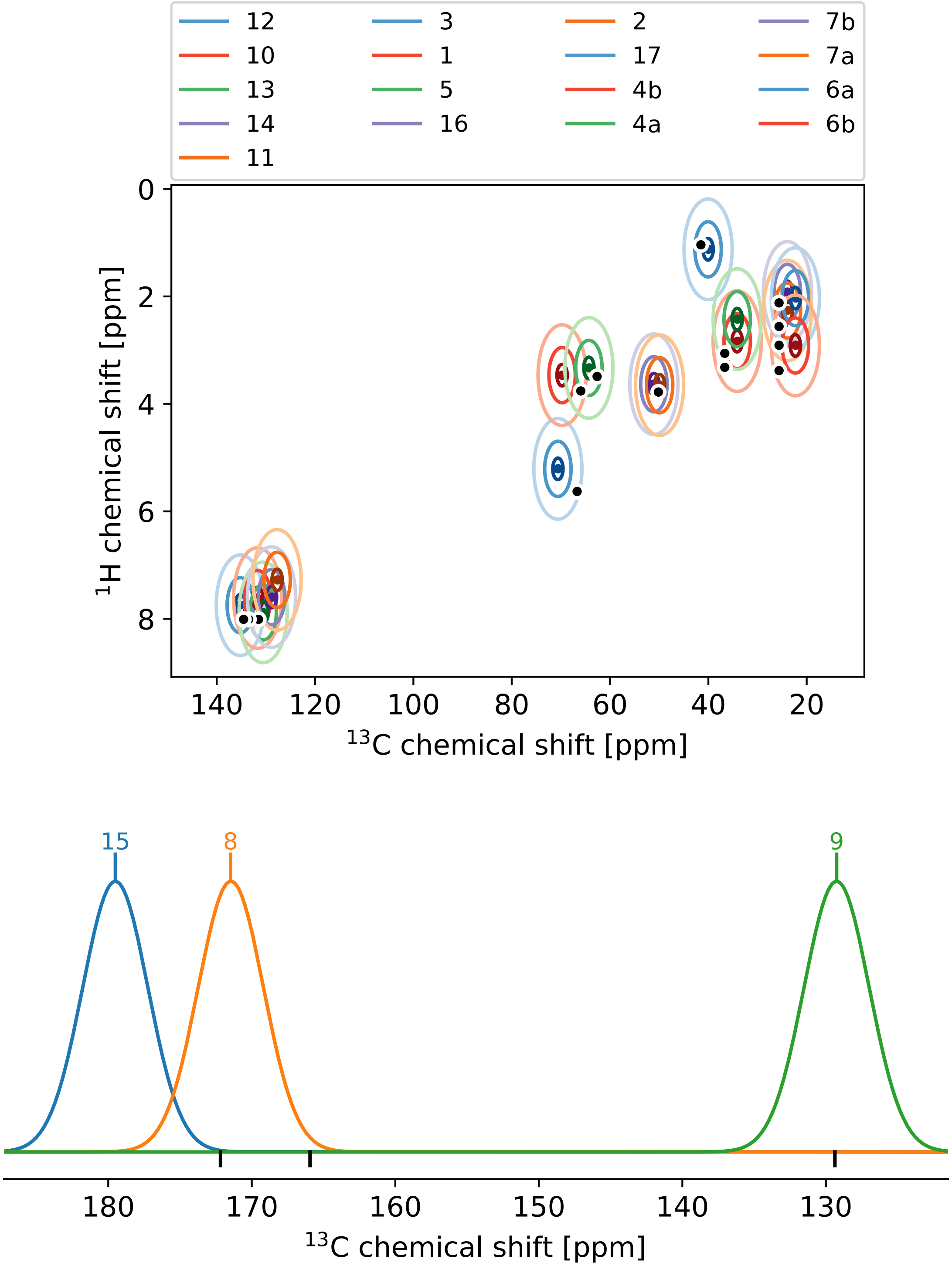

Figure S 13. Chemical shift distributions for cocaine using the XS approach with the DFT shielding predictions. 2D ^{1}H-^{13}C correlated peaks and quaternary ^{13}C spectra, respectively, are colored with the respective labels in the legend, and experimental shifts are indicated by black dots in the correlated ^{1}H-^{13}C chemical shift distributions (upper), as well as by black vertical lines under the 1D ^{13}C chemical shift distributions (lower).

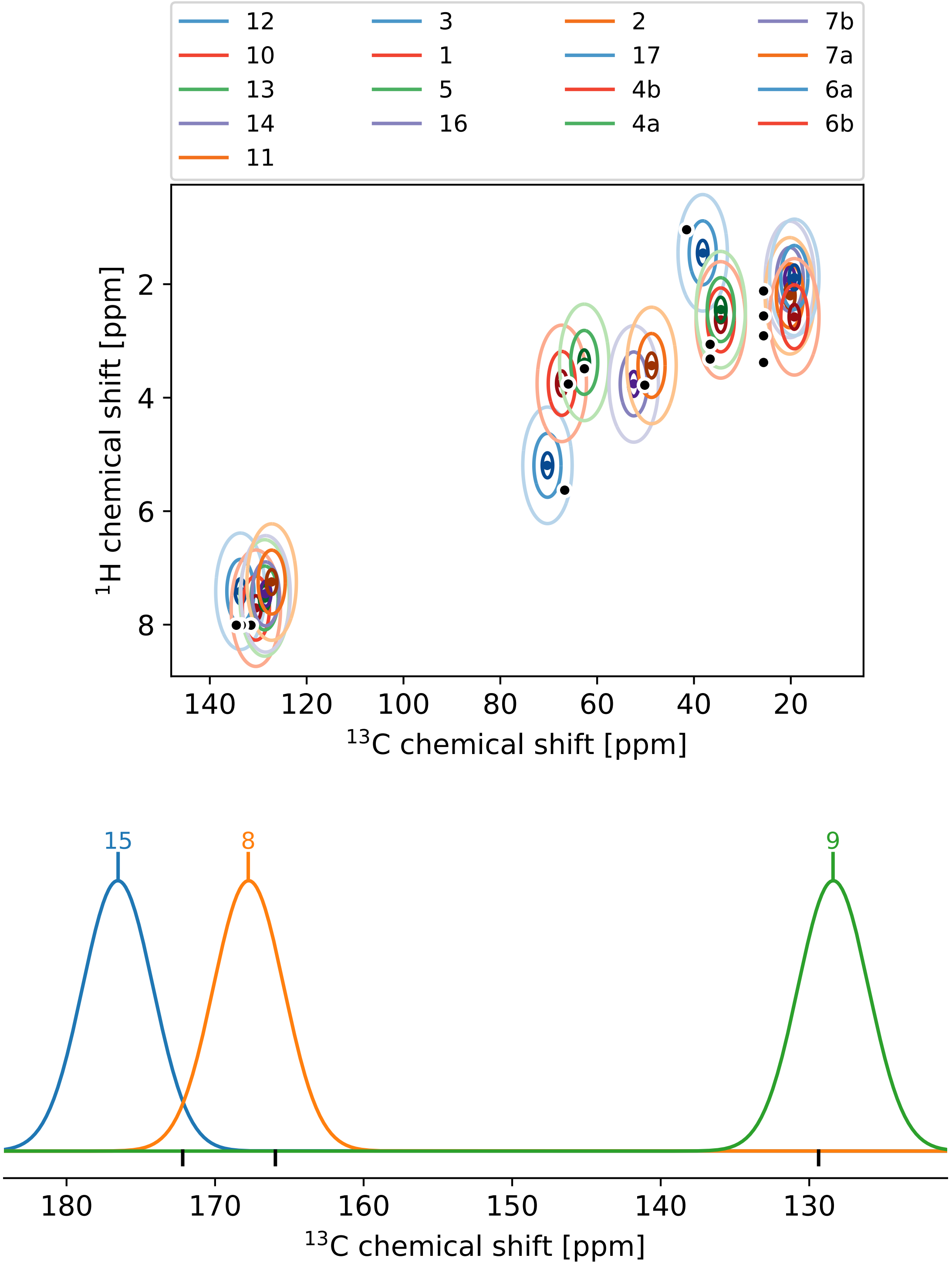

Figure S 14. Chemical shift distributions for cocaine using the XS approach with the ShiftML3 shielding predictions. 2D ^{1}H-^{13}C correlated peaks and quaternary ^{13}C spectra, respectively, are colored with the respective labels in the legend, and experimental shifts are indicated by black dots in the correlated ^{1}H-^{13}C chemical shift distributions (upper), as well as by black vertical lines under the 1D ^{13}C chemical shift distributions (lower).

5.2.2. Atuliflapon

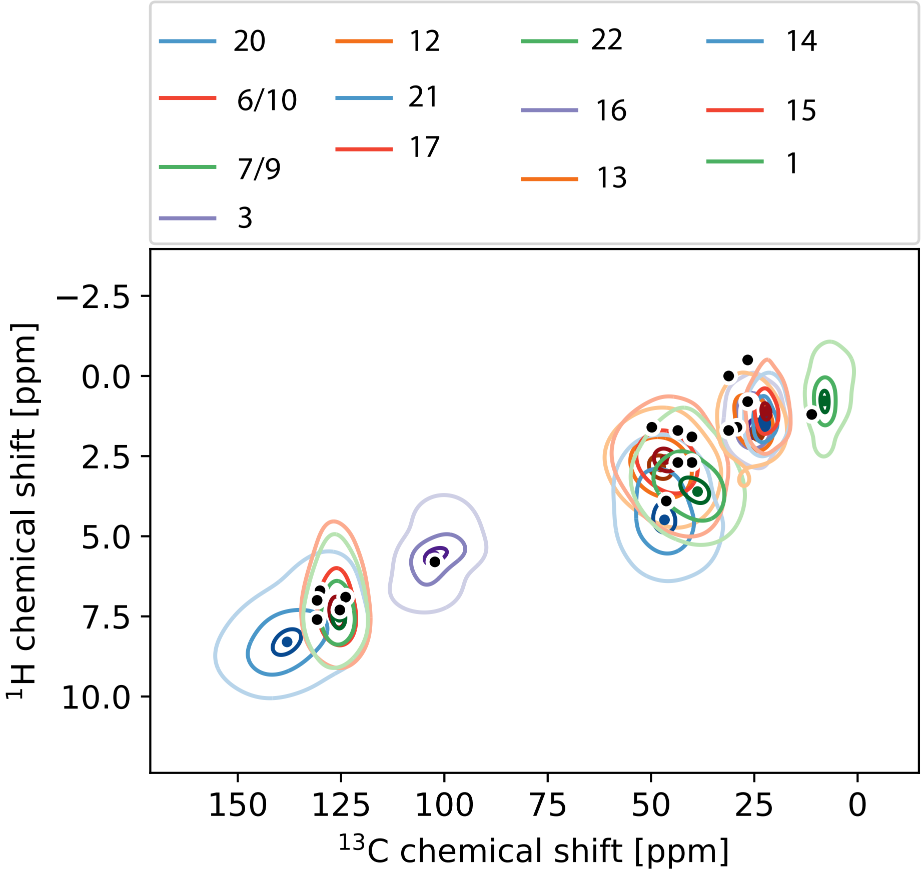

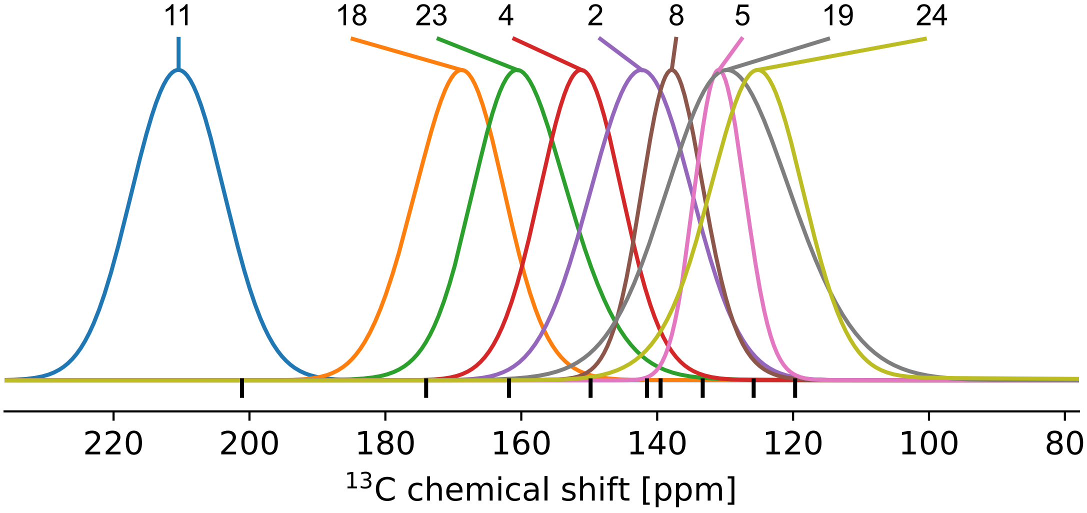

Figure S 15. Chemical shift distributions for Atuliflapon using the DB approach. 2D ^{1}H-^{13}C correlated peaks and quaternary ^{13}C spectra, respectively, are colored with the respective labels in the legend, and experimental shifts are indicated by black dots in the correlated ^{1}H-^{13}C chemical shift distributions (upper), as well as by black vertical lines under the 1D ^{13}C chemical shift distributions (lower).

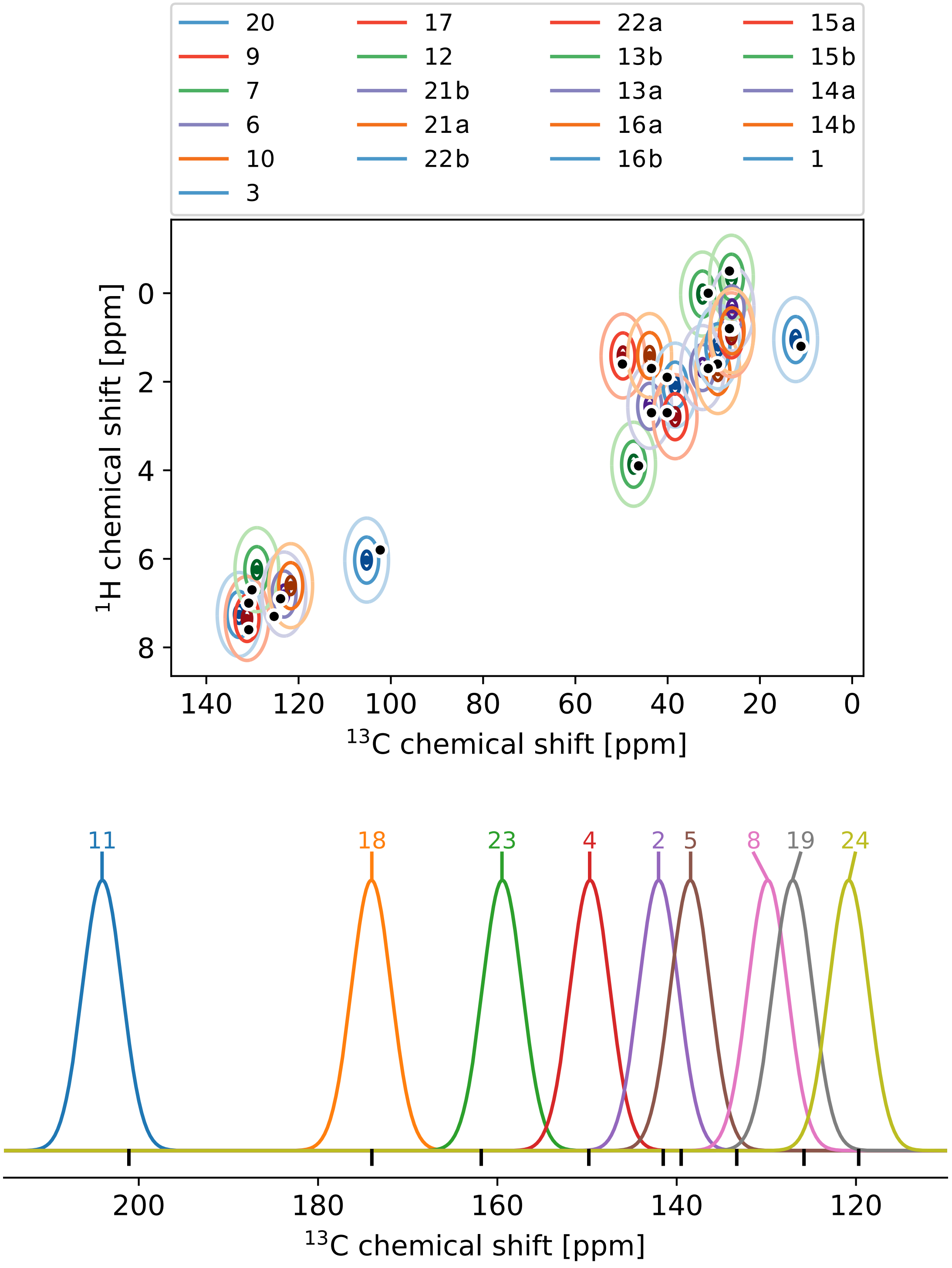

Figure S 16. Chemical shift distributions for Atuliflapon using the XS approach with the DFT shielding predictions. 2D ^{1}H-^{13}C correlated peaks and quaternary ^{13}C spectra, respectively, are colored with the respective labels in the legend, and experimental shifts are indicated by black dots in the correlated ^{1}H-^{13}C chemical shift distributions (upper), as well as by black vertical lines under the 1D ^{13}C chemical shift distributions (lower).

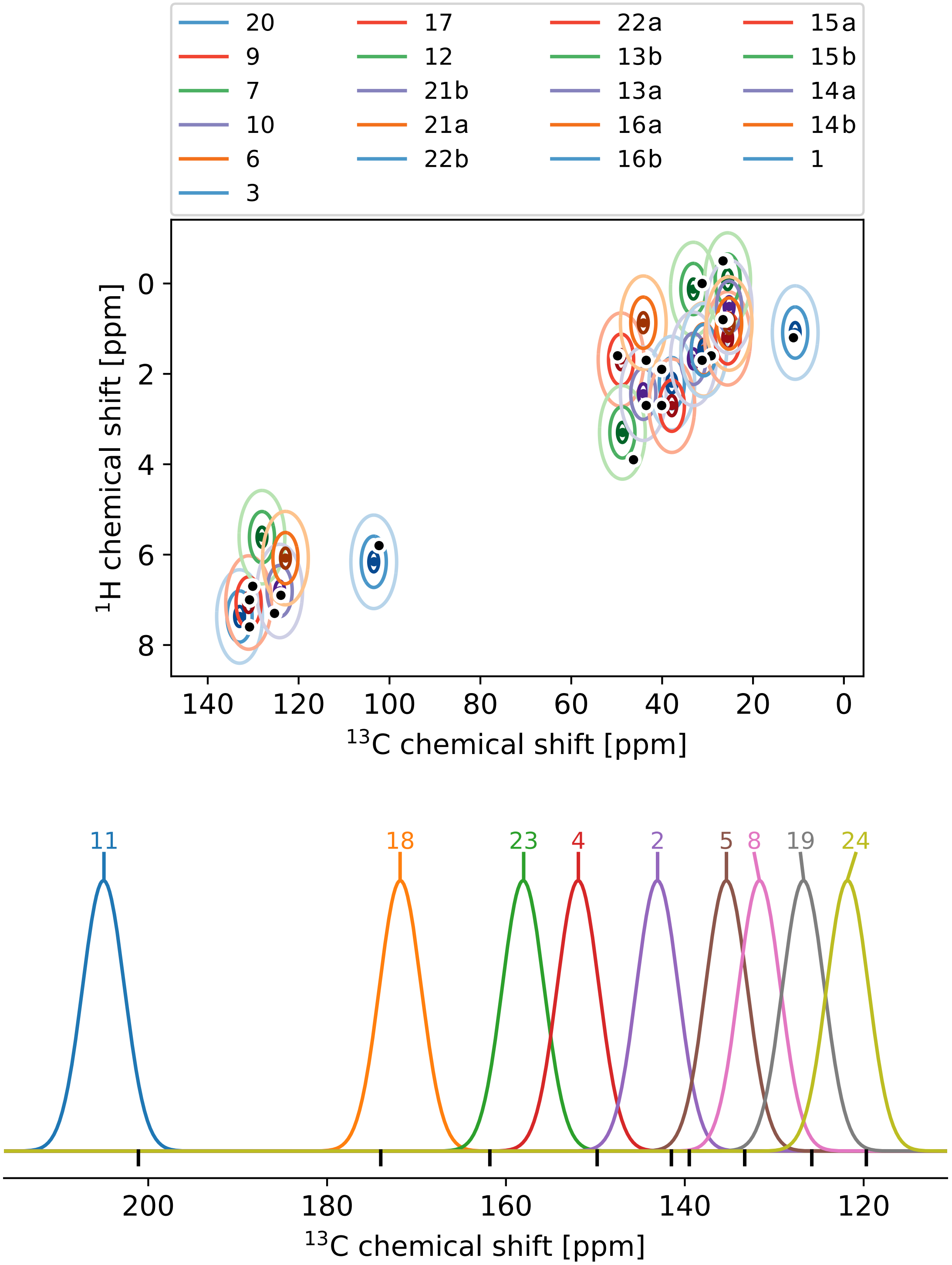

Figure S 17. Chemical shift distributions for Atuliflapon using the XS approach with the ShiftML3 shielding predictions. 2D ^{1}H-^{13}C correlated peaks and quaternary ^{13}C spectra, respectively, are colored with the respective labels in the legend, and experimental shifts are indicated by black dots in the correlated ^{1}H-^{13}C chemical shift distributions (upper), as well as by black vertical lines under the 1D ^{13}C chemical shift distributions (lower).

5.2.3. Lorlatinib

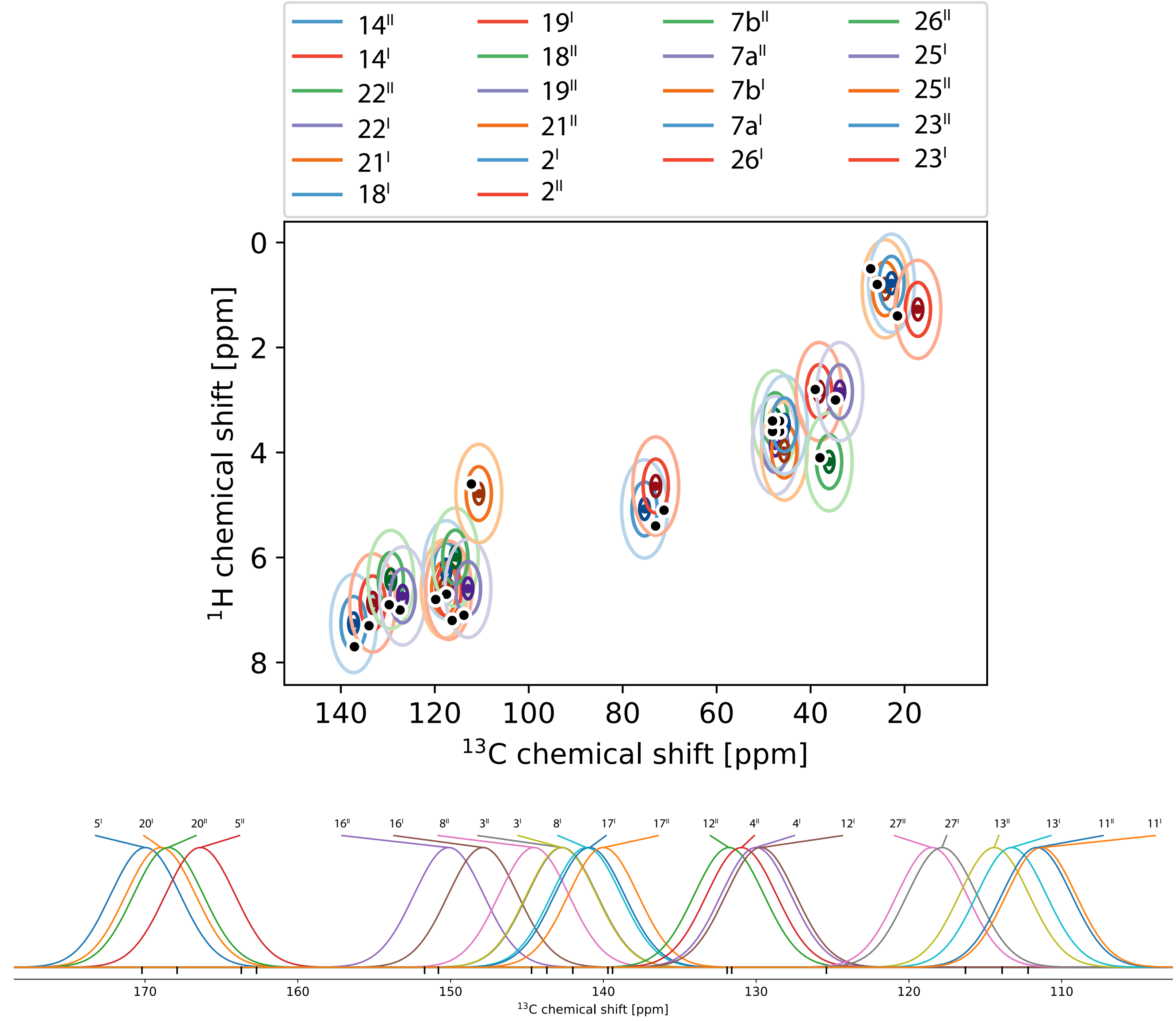

Figure S 18. Chemical shift distributions for Lorlatinib using the XS approach with the DFT shielding predictions. 2D ^{1}H-^{13}C correlated peaks and quaternary ^{13}C spectra, respectively, are colored with the respective labels in the legend, and experimental shifts are indicated by black dots in the correlated ^{1}H-^{13}C chemical shift distributions (upper), as well as by black vertical lines under the 1D ^{13}C chemical shift distributions (lower).

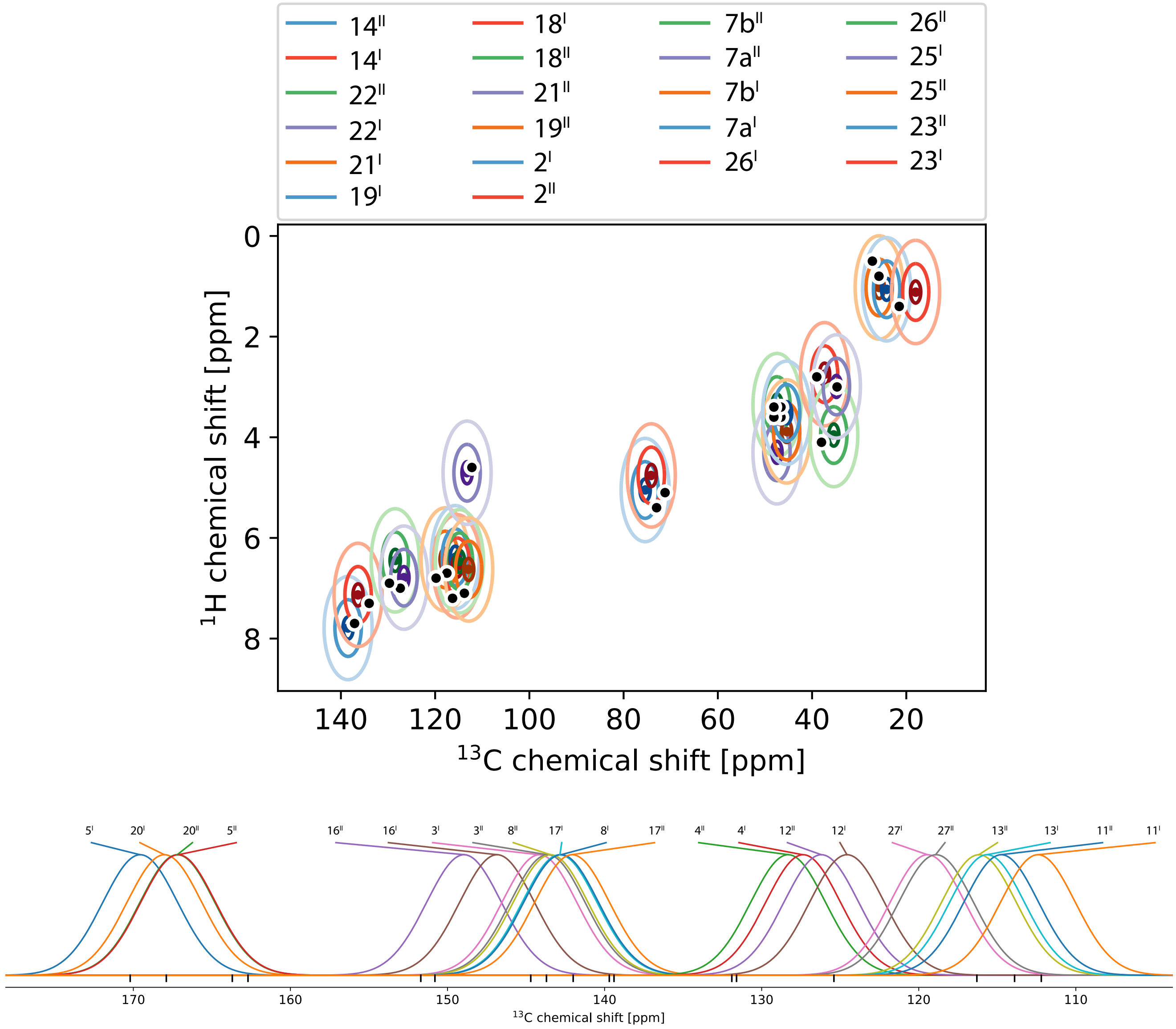

Figure S 19. Chemical shift distributions for Lorlatinib using the XS approach with the ShiftML3 shielding predictions. 2D ^{1}H-^{13}C correlated peaks and quaternary ^{13}C spectra, respectively, are colored with the respective labels in the legend, and experimental shifts are indicated by black dots in the correlated ^{1}H-^{13}C chemical shift distributions (upper), as well as by black vertical lines under the 1D ^{13}C chemical shift distributions (lower).

References

[1] P. Giannozzi, O. Andreussi, T. Brumme, O. Bunau, M. Buongiorno Nardelli, M. Calandra, R. Car, C. Cavazzoni, D. Ceresoli, M. Cococcioni, N. Colonna, I. Carnimeo, A. Dal Corso, S. de Gironcoli, P. Delugas, R. A. DiStasio, Jr., A. Ferretti, A. Floris, G. Fratesi, G. Fugallo, R. Gebauer, U. Gerstmann, F. Giustino, T. Gorni, J. Jia, M. Kawamura, H. Y. Ko, A. Kokalj, E. Kucukbenli, M. Lazzeri, M. Marsili, N. Marzari, F. Mauri, N. L. Nguyen, H. V. Nguyen, A. Otero-de-la-Roza, L. Paulatto, S. Ponce, D. Rocca, R. Sabatini, B. Santra, M. Schlipf, A. P. Seitsonen, A. Smogunov, I. Timrov, T. Thonhauser, P. Umari, N. Vast, X. Wu and S. Baroni, "Advanced capabilities for materials modelling with Quantum ESPRESSO" *J Phys Condens Matter* **2017**, *29*, 465901.

[2] P. Giannozzi, S. Baroni, N. Bonini, M. Calandra, R. Car, C. Cavazzoni, D. Ceresoli, G. L. Chiarotti, M. Cococcioni, I. Dabo, A. Dal Corso, S. de Gironcoli, S. Fabris, G. Fratesi, R. Gebauer, U. Gerstmann, C. Gougoussis, A. Kokalj, M. Lazzeri, L. Martin-Samos, N. Marzari, F. Mauri, R. Mazzarello, S. Paolini, A. Pasquarello, L. Paulatto, C. Sbraccia, S. Scandolo, G. Sclauzero, A. P. Seitsonen, A. Smogunov, P. Umari and R. M. Wentzcovitch, "QUANTUM ESPRESSO: a modular and open-source software project for quantum simulations of materials" *J Phys Condens Matter* **2009**, *21*, 395502.

[3] H. J. Monkhorst and J. D. Pack, "Special points for Brillouin-zone integrations" *Physical Review B* **1976**, *13*, 5188-5192.

[4] J. P. Perdew, in AIP Conference Proceedings, 2001, pp. 1-20.

[5] J. P. Perdew, K. Burke and M. Ernzerhof, "Generalized Gradient Approximation Made Simple" *Phys Rev Lett* **1996**, *77*, 3865-3868.

[6] S. Grimme, "Semiempirical GGA-type density functional constructed with a long-range dispersion correction" *J Comput Chem* **2006**, *27*, 1787-1799.

[7] S. Grimme, J. Antony, S. Ehrlich and H. Krieg, "A consistent and accurate ab initio parametrization of density functional dispersion correction (DFT-D) for the 94 elements H-Pu" *J Chem Phys* **2010**, *132*, 154104.

[8] M. Kellner, J. B. Holmes, R. Rodriguez-Madrid, F. Viscosi, Y. Zhang, L. Emsley and M. Ceriotti, "A deep learning model for chemical shieldings in molecular organic solids including anisotropy" *The Journal of Physical Chemistry Letters* **2025**, *16*, 8714−8722.

[9] M. Baias, C. M. Widdifield, J. N. Dumez, H. P. Thompson, T. G. Cooper, E. Salager, S. Bassil, R. S. Stein, A. Lesage, G. M. Day and L. Emsley, "Powder crystallography of pharmaceutical materials by combined crystal structure prediction and solid-state ^{1}H NMR spectroscopy" *Phys Chem Chem Phys* **2013**, *15*, 8069-8080.

[10] M. Cordova, M. Balodis, B. Simoes de Almeida, M. Ceriotti and L. Emsley, "Bayesian probabilistic assignment of chemical shifts in organic solids" *Sci Adv* **2021**, *7*, eabk2341.

[11] M. Cordova, M. Balodis, A. Hofstetter, F. Paruzzo, S. O. Nilsson Lill, E. S. E. Eriksson, P. Berruyer, B. Simoes de Almeida, M. J. Quayle, S. T. Norberg, A. Svensk Ankarberg, S. Schantz and L. Emsley, "Structure determination of an amorphous drug through large-scale NMR predictions" *Nat Commun* **2021**, *12*, 2964.

[12] Z. Rehman, W. T. Franks, B. Nguyen, H. F. Schmidt, G. Scrivens and S. P. Brown, "Discovering the Solid-State Secrets of Lorlatinib by NMR Crystallography: To Hydrogen Bond or not to Hydrogen Bond" *J Pharm Sci* **2023**, *112*, 1915-1928.
